# Arabidopsis KNS3 and its two homologs mediate endoplasmic reticulum-to-plasma membrane traffic of boric acid channels

**DOI:** 10.1093/jxb/erae380

**Published:** 2024-10-30

**Authors:** Zhe Zhang, Shunsuke Nakamura, Arisa Yamasaki, Masataka Uehara, Shunsuke Takemura, Kohei Tsuchida, Takehiro Kamiya, Shuji Shigenobu, Katsushi Yamaguchi, Toru Fujiwara, Sumie Ishiguro, Junpei Takano

**Affiliations:** Graduate School of Life and Environmental Sciences, Osaka Prefecture University, 1-1 Gakuen-cho, Naka-ku, Sakai, Osaka, 599-8531, Japan; Graduate School of Agriculture, Hokkaido University, Kita 8, Nishi 5, Kita-ku, Sapporo, Hokkaido, 060-0808, Japan; Graduate School of Life and Environmental Sciences, Osaka Prefecture University, 1-1 Gakuen-cho, Naka-ku, Sakai, Osaka, 599-8531, Japan; Graduate School of Agriculture, Hokkaido University, Kita 8, Nishi 5, Kita-ku, Sapporo, Hokkaido, 060-0808, Japan; Graduate School of Bioagricultural Sciences, Nagoya University, Furo-cho, Chikusa-ku, Nagoya, 464-8601, Japan; Graduate School of Bioagricultural Sciences, Nagoya University, Furo-cho, Chikusa-ku, Nagoya, 464-8601, Japan; Graduate School of Agricultural and Life Sciences, The University of Tokyo, 1-1-1 Yayoi, Bunkyo-ku, Tokyo, 113-8657, Japan; National Institute for Basic Biology, 38 Nishigonaka, Myodaiji-cho, Okazaki, 444-8585, Japan; National Institute for Basic Biology, 38 Nishigonaka, Myodaiji-cho, Okazaki, 444-8585, Japan; Graduate School of Agricultural and Life Sciences, The University of Tokyo, 1-1-1 Yayoi, Bunkyo-ku, Tokyo, 113-8657, Japan; Graduate School of Bioagricultural Sciences, Nagoya University, Furo-cho, Chikusa-ku, Nagoya, 464-8601, Japan; Graduate School of Life and Environmental Sciences, Osaka Prefecture University, 1-1 Gakuen-cho, Naka-ku, Sakai, Osaka, 599-8531, Japan; Graduate School of Agriculture, Osaka Metropolitan University, 1-1 Gakuen-cho, Naka-ku, Sakai, Osaka, 599-8531, Japan; Cardiff University, UK

**Keywords:** *Arabidopsis thaliana*, boric acid channel, cargo receptor complex, endoplasmic reticulum exit, KNS3/SPOT1/IEF, membrane trafficking

## Abstract

Membrane proteins targeted to the plasma membrane are first transported from the endoplasmic reticulum (ER) to the Golgi apparatus. This study explored the mechanisms controlling plasma membrane trafficking of the boric acid channel AtNIP5;1 from the ER. Imaging-based screening using transgenic Arabidopsis identified six mutants in which GFP-NIP5;1 was localized in the ER in addition to the plasma membrane. Genetic mapping and whole-genome resequencing identified the responsible gene in four among the six mutants as *KAONASHI3 (KNS3)/SPOTTY1/IMPERFECTIVE EXINE FORMATION*. Among the plasma membrane-localized proteins tested, NIP5;1 and its homolog NIP6;1 were retained in the ER of the *kns3* mutants. Our genetic analysis further discovered that two homologs of KNS3, KNSTH1 and KNSTH2, were also involved in the ER exit of NIP5;1. In Arabidopsis protoplasts and tobacco leaves, mCherry-fused KNS3 localized to the ER and Golgi, whereas KNSTH2 localized to the ER. The cytosolic C-terminal tail of KNS3 contains amino acids important for Golgi-to-ER trafficking. Furthermore, the ER-to-Golgi trafficking of KNS3 depended on KNSTH1 and KNSTH2, and the accumulation of these three proteins in Arabidopsis roots depended on each other. We propose that KNS3, KNSTH1, and KNSTH2 function as a cargo-receptor complex mediating the ER exit of NIP5;1.

## Teaser

KAONASHI3 (KNS3)/SPOTTY1/IMPERFECTIVE EXINE FORMATION and its two homologs function cooperatively in the endoplasmic reticulum-to-plasma membrane traffic of boric acid channels as a potential cargo-receptor complex in *Arabidopsis thaliana* .

## Introduction

Membrane proteins destined for the plasma membrane (PM) are co-translationally inserted into the membrane of the endoplasmic reticulum (ER) and then transported through the Golgi stacks and the *trans*-Golgi network to the PM. Trafficking between the ER and Golgi apparatus involves Coat Protein I (COPI)- and COPII-coated vesicles. COPI-coated vesicles mediate retrograde trafficking between the Golgi stacks and *cis*-Golgi to the ER, whereas COPII-coated vesicles mediate trafficking from the ER exit sites to the Golgi. COPII comprises five proteins, SAR1, SEC23, SEC24, SEC13, and SEC31. Among these components, SEC24 directly interacts with the signals exposed on the cytosolic side of cargo proteins. This behavior makes SEC24 an important factor in cargo selection for COPII vesicles (Béthune and Wieland, 2018; Brandizzi, 2018). Cargo proteins do not necessarily have signals that are recognized by SEC24. Cargo receptors interact with cargo proteins and SEC24 to facilitate the ER exit of the cargo proteins (Gomez-Navarro and Miller, 2016; Béthune and Wieland, 2018).

In plants, there is limited information on the cargo receptors for trafficking between the ER and Golgi apparatus. p24 proteins interact with the K/HDEL receptor ER RETENTION DEFECTIVE2 and facilitate the retrograde transport of ER RETENTION DEFECTIVE2 and K/HDEL ligands from the Golgi apparatus to the ER in *Arabidopsis thaliana* (Montesinos *et al.*, 2014; Pastor-Cantizano *et al.*, 2017, 2018). CORNICHON HOMOLOG (CNIH)1 functions as a possible cargo receptor for trafficking the sodium transporter OsHKT1;3 from the ER to the Golgi apparatus in rice (*Oryza sativa*) (Rosas-Santiago *et al.*, 2015, 2017). AtCNIH1 and AtCNIH4 are essential for sorting and trafficking Glutamate Receptor-like3.3 from the ER to the PM in Arabidopsis (Wudick *et al.*, 2018). PpCNIH2 functions as a cargo receptor important for ER exit and polar localization of the auxin efflux transporter PINA in the protonema cells of the moss *Physcomitrium patens* (Yáñez-Domínguez *et al.*, 2023).

Boron (B) is an essential element for plant growth and is necessary for the structure and function of cell walls by cross-linking pectin in the rhamnogalacturonan II region (Funakawa and Miwa, 2015). Nodulin 26-like Intrinsic Protein (NIP)5;1 is a boric acid channel localized in the PM of root cells, including epidermal and endodermal cells. NIP5;1 is involved in B uptake under low-B conditions. Arabidopsis *NIP5;1* T-DNA insertion mutants show severely reduced root and shoot growth owing to defects in B uptake under low-B conditions (Takano *et al.*, 2006). NIP5;1 shows polar localization in the PM toward the soil side in root epidermal and endodermal cells, and this polar localization is important for the efficient transport of B in roots (Alassimone *et al.*, 2010; Takano *et al.*, 2010; Wang *et al.*, 2017). NIP5;1 belongs to the NIP subfamily of major intrinsic proteins (aquaporins; Maurel *et al.*, 2015; Jothi and Takano, 2023). In the NIP subfamily, there are two other boric acid channels, NIP6;1 and NIP7;1, which are important for B transport under low-B conditions. NIP6;1 is expressed in the nodal regions of shoots, especially in the phloem region of vascular tissues, and is required for the distribution of B toward young developing shoot tissues under low-B conditions. T-DNA insertion mutants of *NIP6;1* exhibited reduced expansion of young rosette leaves under low-B conditions (Tanaka *et al.*, 2008). NIP7;1 is expressed in the developing anthers and is involved in pollen development (Li *et al.*, 2011). Compared with observations in the wild type (WT), T-DNA insertion mutants of *NIP7;1* exhibited shorter siliques, a higher number of aborted seeds, and morphological defects in pollen grains under low-B conditions (Routray *et al.*, 2018).

In previous work, we screened Arabidopsis mutants in which GFP-NIP5;1 showed aberrant localization and identified six mutant lines showing ER localization of GFP-NIP5;1 (Uehara *et al.*, 2014). In the present study, we aimed to use these mutants to understand the mechanism underlying the ER exit of boric acid channels. Using genetic mapping and whole-genome resequencing, we identified *KAONASHI3 (KNS3)/SPOTTY1/IMPERFECTIVE EXINE FORMATION* and its homologs as the genes responsible for the mutant phenotype. We showed that KNS3 is localized in the ER and Golgi, and identified three amino acids in the C-terminal tail of KNS3 important for its Golgi-to-ER trafficking. We also showed that two homologs of KNS3 are important for ER-to-Golgi trafficking of KNS3 and that the accumulation of these proteins is dependent on each other. We propose that KNS3 and its two homologs function as a cargo-receptor complex for the ER exit of boric acid channels.

## Materials and methods

### Plant materials and growth conditions

*Arabidopsis thaliana* ecotypes Col-0 and *Ler* were from our laboratory stocks. The T-DNA insertion mutants *kns3-2* (SALK_041228), *kns3-3* (SALK_061320), *knsth1-1* (SALK_027378), *knsth1-2* (SALK_106609), *knsth2-1* (SAIL_731_H03), and *knsth2-3* (SAIL_670_H01) were obtained from the Arabidopsis Biological Resource Center. The *kns3-2 knsth1-1*, *kns3-2 knsth2-1*, and *knsth1-1 knsth2-1* double mutants, and the *kns3-2 knsth1-1 knsth2-1* triple mutant were generated by crossing. The *nip5;1-1* mutant (SALK_122287) and ProNIP5;1 (–5ʹ untranslated regions):GFP-NIP5;1/*nip5;1-1* transgenic plants were described previously (Takano *et al.*, 2006; Tanaka *et al.*, 2011).

Seeds were surface sterilized with 1% (w/v) NaClO for 10 min and rinsed with ultrapure water. For imaging of GFP-NIP5;1, plants were grown on solid medium [1.51 mM NaH_2_PO_4_, 0.26 mM Na_2_HPO_4_, 1.5 mM MgSO_4_, 2.0 mM Ca(NO_3_)_2_, 3.0 mM KNO_3_, 10.3 μM MnSO_4_, 1.0 μM ZnSO_4_, 1.0 μM CuSO_4_, 130 nM CoCl_2_, 24 nM (NH_4_)_6_Mo_7_O_24_, and 50 μM FeNa-EDTA] (Takano *et al.*, 2005) containing 1% (w/v) sucrose, 1% (w/v) gellan gum, and 30 µM B for 4–10 d in growth chambers at 22 °C under fluorescent lamps (approximately 90 µmol m^–2^ s^–1^) with long-day conditions (16 h/8 h light/dark cycle). For phenotypic analysis and B determination in the reproductive growth stage, plants were cultivated in a hydroponic culture system (Takano *et al.*, 2001) with liquid medium (Takano *et al.*, 2005) containing 30 μM B for 10–12 d and then transferred to liquid medium containing 1 μM or 100 μM B for a further 15–30 d of growth under fluorescent lamps (approximately 110 µmol m^–2^ s^–1^) with long-day conditions. To observe pollen grains, plants were grown on Rockwool and vermiculite in pots and supplied with 1/1000 diluted Hyponex solution (Hyponex, Japan) once a week.

### Genetic mapping and whole-genome resequencing

For genetic mapping, lines 1–3, 10–6, 14–3, and 15–2 (Col-0 background) were crossed with the *Ler* ecotype to obtain selfed F_2_ seeds. Genomic DNA was extracted from F_2_ plants exhibiting a mutant phenotype of GFP-NIP5;1. For rough mapping, simple sequence length polymorphism markers from the TAIR marker database and Cereon database (http://www.arabidopsis.org) were used as follows: map1-3M, map1-13.8M, and map1-24.4M for chromosome 1; map2-3.5M and nga168 for chromosome 2; nga162 and map3-18M for chromosome 3; NGA8 for chromosome 4; 2.5M and map5-19.9M for chromosome 5 (Supplementary Table S1). The whole-genome resequencing method has been described previously (Uehara *et al.*, 2014).

### Subcellular localization

The coding sequences of *mCherry*, *KNS3*, and *KNSTH2* were amplified by PCR using specific primers (Supplementary Table S2). The PCR products were assembled using the In-Fusion technique (Clontech) into the pUB-DEST vector (Grefen *et al.*, 2010) digested by *Xho*I for agroinfiltration into *Nicotiana benthamiana* (tobacco) leaves or into the pUGW2C vector (Nishimura *et al.*, 2015) digested by *Pme*I and *Spe*I for polyethylene glycol (PEG)-mediated transformation into Arabidopsis mesophyll protoplasts. Alanine substitutions in the C-terminal regions of KNS3 and KNSTH2 were performed by inverse PCR using specific primers and vectors containing mCherry-KNS3 and mCherry-KNSTH2 as templates (Supplementary Table S2). The ends of the linear PCR products were fused using the In-Fusion technique. The 35S promoter:ST-YFP plasmid (Ito *et al.*, 2018) was provided by Yoko Ito. The 2 × 35S promoter:Man1-GFP and GFP-HDEL plasmids (G-gb and ER-gb; Nelson *et al.*, 2007) were provided by the Arabidopsis Biological Resource Center.

PEG-mediated protoplast transformation was performed as described previously (Maekawa *et al.*, 2014). Agroinfiltration was performed as described previously (Yasuda *et al.*, 2014) with some modifications. *Agrobacterium* was transformed with constructs using the heat shock method and then grown in Yeast Extract Peptone medium. The *Agrobacterium* cells were collected and resuspended with infiltration buffer containing 150 µM acetosyringone.

Immunofluorescence was performed as previously described (Yoshinari *et al.*, 2019) with a few modifications. The specimens were incubated with the primary antibody [rabbit polyclonal anti-binding immunoglobulin protein (BiP) antibody (1:6000, Agrisera), chicken polyclonal anti-PIN FORMED 2 (PIN2) antibody (1:1000, Agrisera), rabbit polyclonal anti-PENTRATION3 (PEN3) antibody (1:1000, Agrisera), and rabbit polyclonal anti-PM intrinsic protein 2 (PIP2) antibody (1:1000, Agrisera) diluted in the blocking buffer] and secondary antibody [goat anti-rabbit IgG antibody conjugated with DyLight 549 (1:500; Thermo Fisher Scientific) and goat anti-chicken IgG antibody conjugated with CF568 (1:1000; Biotium)]. The specimens were counterstained with DAPI (2 μg ml^–1^) for 20 min at room temperature and washed four times with ultrapure water. Finally, the specimens were mounted using SlowFade Gold antifade solution (Thermo Fisher Scientific).

Confocal image acquisition was performed using a Leica TCS SP8 equipped with a ×40 water-immersion lens or a ×63 oil-immersion lens. The laser excitation/spectral detection bandwidths were 405/420–470 nm for DAPI; 488/500–530 nm for GFP; 488/520–550 nm for YFP; 552/580–650 nm for mCherry, DyLight 549, and CF 568; and 552/650–700 nm for FM4-64. The image contrast was adjusted using ImageJ and Fiji software (Schindelin *et al.*, 2012). Pearson correlation coefficients were calculated using Fiji/ImageJ with the PSC co-localization plugin (French *et al.*, 2008).

### Measurement of boron concentration in plant tissues

The roots, rosette leaves, and shoot apices (~1.5 cm from the top of the plants) were harvested from three or four independent plants grown hydroponically under long-day conditions. The samples were dried, weighed, and digested using concentrated nitric acid (FUJIFILM Wako Chemicals, Osaka, Japan) for B determination. The samples were dissolved in 0.3 M nitric acid. The B concentration was determined using a curcumin assay (Mohan and Jones, 2018).

### Preparation of microsomal proteins and western blot analysis

The anti-KNS3, anti-KNSTH1, and anti-KNSTH2 antibodies were purchased from Sigma-Aldrich. Peptides [CNDHTSLKGGHAHS for KNS3 (68% purity), CALSGDGVLPRGEFHPLAA for KNSTH1 (80% purity), CSAPYEKTSHAHERPITN for KNSTH2 (85% purity)] were synthesized and used to immunize rabbits. Eight weeks after immunization, approximately 60 ml of serum was collected and affinity purified using these peptides.

Preparation of microsomal proteins and western blotting were performed as previously described (Yoshinari *et al.*, 2019) with a few modifications. Root tissues (0.2–0.5 g) were lysed in 2–3 ml of homogenization buffer. Protein samples were heated at 70 °C for 10 min in SDS sample buffer containing 10% (v/v) 2-mercaptoethanol. Proteins were loaded on to a Bolt 4–12% Bis-Tris gel (Thermo Fisher Scientific) and transferred to a polyvinylidene fluoride membrane using a semi-dry transfer technique (Trans-Blot system; Bio-Rad). Rabbit anti-KNS3 polyclonal antibody (1:5000), rabbit anti-KNSTH1 polyclonal antibody (1:2000), and rabbit anti-KNSTH2 polyclonal antibody (1:5000) were diluted in Can Get Signal Solution 1 (Toyobo) and used as primary antibodies. An anti-rabbit IgG antibody conjugated with horseradish peroxidase (Jackson ImmunoResearch) and diluted at 1:1 000 000 in Can Get Signal Solution 2 (Toyobo) was used as the secondary antibody. Protein signals were detected using a chemiluminescence imaging system (FUSION SOLO.7S.EDGE; Vilber, Germany). The membrane-bound proteins were stained with Coomassie Brilliant Blue (ATTO) after detection.

### Statistical analysis

When data were found to be normally distributed, either Dunnett’s or Tukey’s multiple comparison test was conducted. When data were found not to be normally distributed, a non-parametric test (Mann–Whitney U test) was used. Prism 8 software (version 8.4.2; GraphPad Software, San Diego, CA, USA) was used for these analyses.

### Multiple sequence alignment and phylogenetic analysis

Multiple sequence alignments of KNS3 and its orthologous amino acid sequences were performed using Clustal Omega (EMBL-EBI), and a phylogenetic tree was constructed using MEGA X (Kumar *et al.*, 2018). The reliability of the topology was examined using the bootstrap method, which generated a bootstrap probability of 1000 replications at each interior branch of the tree.

## Results

### Mutations in *KNS3* cause defects in the endoplasmic reticulum exit of NIP5;1

**Fig. 1.**
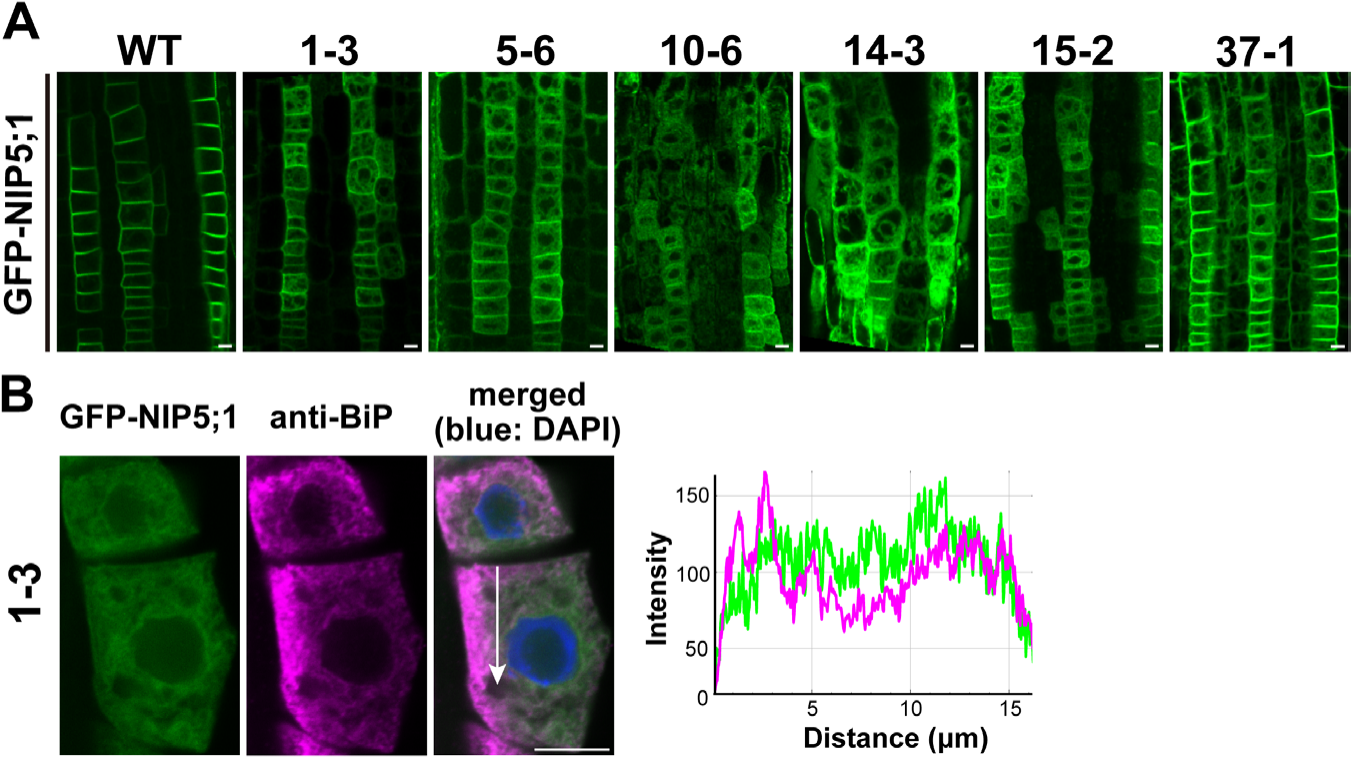
Identification of mutants in which GFP-NIP5;1 was localized at the endoplasmic reticulum and plasma membrane. (A) GFP-NIP5;1 in the root epidermal cells of a wild-type (WT) plant and ethyl methanesulfonate mutants. (B) Left panel: immunofluorescence in root epidermal cells of line 1–3. GFP-NIP5;1 (green), endogenous binding immunoglobulin protein (BiP) detected by anti-BiP antibody (magenta), and nuclei stained with 2 μg ml‐ DAPI (blue) are shown. Right panel: intensity profiles of GFP-NIP5;1 (green) and anti-BiP (magenta), obtained using ImageJ software, along a line (white arrow) in the merged image. Plants were grown on solid medium containing 30 μM B for 7–10 d. Scale bar=10 μm.

NIP5;1 is localized in the PM of the root cap and in root epidermal and endodermal cells in a polar manner toward the soil side (Takano *et al.*, 2010). To understand the intracellular transport mechanism of NIP5;1, we performed genetic screening using an ethyl methanesulfonate-mutagenized population of GFP-NIP5;1 transgenic Arabidopsis plants (*proNIP5;1* (*Δ5ʹ untranslated regions*):*GFP-NIP5;1* in *nip5;1-1*). We screened approximately 40 000 M_2_ seedlings using a fluorescence microscope and identified three mutant lines showing GFP-NIP5;1 intracellular aggregation (Uehara *et al.*, 2014). Additionally, we identified six mutant lines (1–3, 5–6, 10–6, 14–3, 15–2, and 37–1) in which GFP-NIP5;1 showed a network-like distribution in the cytoplasm in addition to localization in the PM of the root epidermal cells (Fig. 1A). To test for allelism, we crossed six mutant lines. In the F_1_ plants of line 1–3 × line 10–6, line 1–3 × line 15–2, line 10–6 × line 14–3, line 10–6 × line 15–2, and line 14–3 × line 15–2, GFP-NIP5;1 was localized in the ER and PM (Supplementary Fig. S1A). However, in the F_1_ plants of line 1–3 × line 37–1, line 5–6 × line 15–2, line 5–6 × line 37–1, and line 10–6 × line 37–1, GFP-NIP5;1 was localized only in the PM. These results suggested that lines 1–3, 10–6, 14–3, and 15–2 were allelic to each other and lines 5–6 and 37–1 were not allelic with these four lines.

To confirm the localization of GFP-NIP5;1 in lines 1–3, 10–6, 14–3, and 15-2, we performed immunofluorescence using an antibody against an ER-resident protein, BiP (Benghezal *et al.*, 2000). BiP was distributed as a network in the cytoplasm, and line plot analysis showed an overlap with GFP-NIP5;1 in line 1–3 (Fig. 1B). The ER localization, in addition to PM localization, indicated that GFP-NIP5;1 was partially retained in the ER of the mutant lines.

Next, to identify the causative gene of the mutant phenotype in lines 1–3, 10–6, 14–3, and 15–2, we performed genetic mapping and whole-genome sequencing. We outcrossed these four mutants (Col-0 background) with the *Ler* ecotype and analyzed the phenotypes of the F_2_ plants. Approximately 25% of the population showed ER retention of GFP-NIP5;1, indicating that the phenotype was caused by a single recessive mutation. Genotyping of 25–35 F_2_ plants showing ER retention with 10 simple sequence length polymorphism markers showed a linkage to a marker located on the lower arm of chromosome 5 (Supplementary Fig. S1B; Supplementary Table S1).

**Fig. 2.**
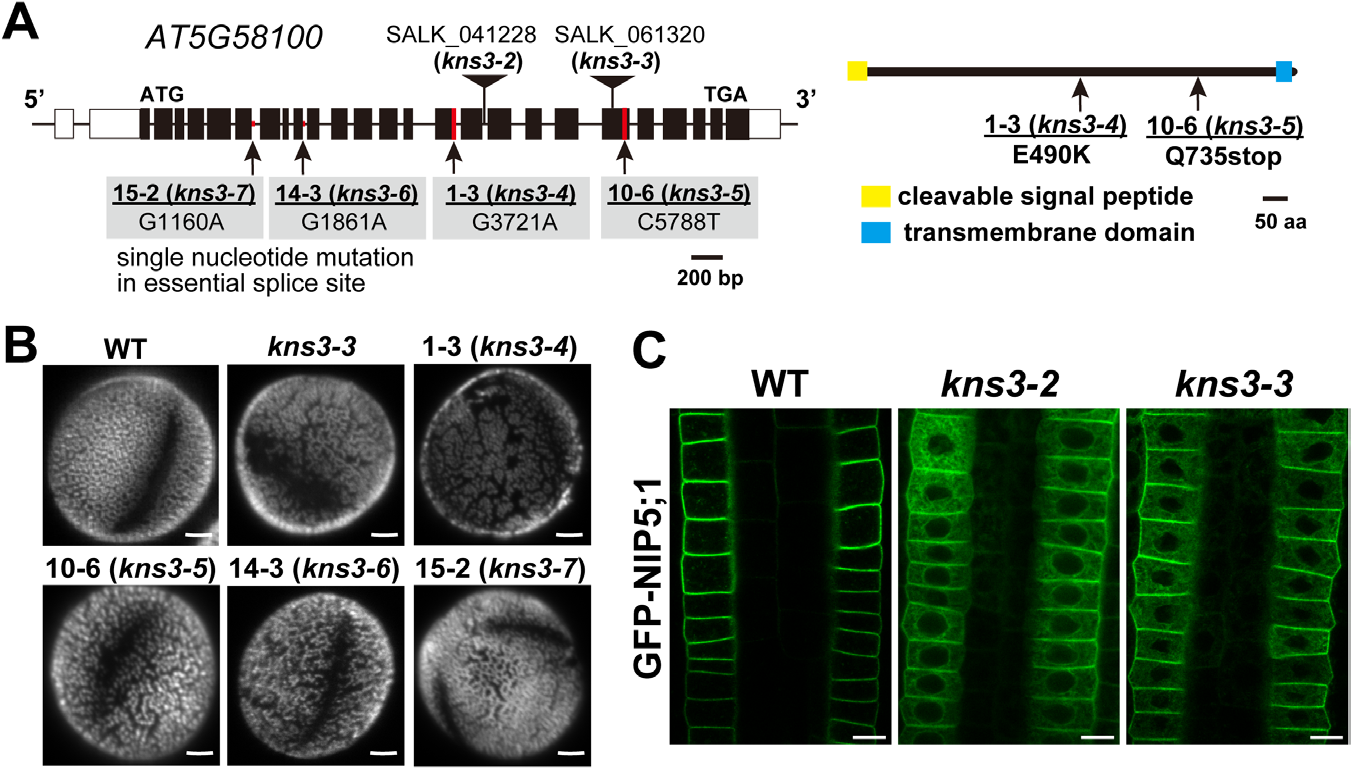
*At5G58100* (*KNS3*) is the causative gene for mutants with defective NIP5;1 localization. (A) The exon–intron structure of the *KNS3* gene (*At5G58100*). Filled boxes, open boxes, and thick bars indicate exons, untranslated regions, and introns, respectively. The topology of KNS3 was predicted by the Philius program. Positions for T-DNA insertion in the *kns3-2* and *kns3-3* mutants and point mutations in line 1–3 (*kns3-4*), 10–6 (*kns3-5*), 14–3 (*kns3-6*), and 15–2 (*kns3-7*) are indicated. (B) Pollen grains of wild-type (WT) plants and *kns3* mutants. Pollen grains were stained with 0.001% Auramine O. Plants were grown in pots with vermiculite supplied with 1/1000 diluted Hyponex solution. (C) GFP-NIP5;1 in the root epidermal cells of *KNS3* T-DNA insertion mutants. Plants were grown on solid medium containing 30 μM B for 7–10 d. Scale bars=2.5 μm (B) and 10 μm (C).

We performed whole-genome sequencing of the four lines using the SOliD platform (Applied Biosystems). The read sequences were mapped to the *A. thaliana* Col-0 genome (TAIR10.0). Single-nucleotide polymorphisms (SNPs) 6420, 3489, 8753, and 9239 were identified in lines 1–3, 10–6, 14–3, and 15–2, respectively. Among the genes containing SNPs, only *At5g58100* was selected based on the following criteria: the type of SNP was (i) C to A or G to T, which are typical substitutions caused by ethyl methanesulfonate, (ii) located on chromosome 5, and (iii) common in the four allelic lines. We identified a missense mutation of the glutamic acid codon to a lysine codon (E490K) in exon 15 in line 1–3, a glutamine codon to a stop codon (Q735stop) in exon 20 in line 10–6, and a single nucleotide substitution in the essential splice site (G/A) at exon 6 downstream in line 15–2 and at exon 9 downstream in line 14–3 (Fig. 2A).

Previously, loss-of-function mutants of *At5g58100* have been reported as *kns3* (Suzuki *et al.*, 2008), *spotty1* (Dobritsa *et al.*, 2011), and *imperfective exine formation* (Wang *et al.*, 2021), which have defects in outer pollen wall formation. To confirm that *At5g58100* is the causative gene for ER retention of NIP5;1, we acquired *kns3-2* and *kns3-3* T-DNA insertion lines (Fig. 2A) and compared their phenotypes. As expected, Auramine O staining showed a collapsed reticulate exine structure on the pollen surface in all four allelic lines and *kns3-3* (Fig. 2B). We then introduced the GFP-NIP5;1 construct into the *kns3-2* and *kns3-3* mutants and observed the fluorescence of GFP-NIP5;1 in root epidermal cells. GFP-NIP5;1 was localized in the ER and PM of the *kns3-2* and *kns3-3* mutants (Fig. 2C), in a pattern similar to that observed in the four allelic lines (Fig. 1A). Collectively, these results establish that *At5g58100* is the causative gene for ER retention of NIP5;1. The four allelic lines, 1–3, 10–6, 14–3, and 15–2, were named *kns3-4*, *kns3-5*, *kns3-6*, and *kns3-7*, respectively.

### KNS3 functions in the endoplasmic reticulum exit of specific plasma membrane proteins, including NIP5;1 and NIP6;1

**Fig. 3.**
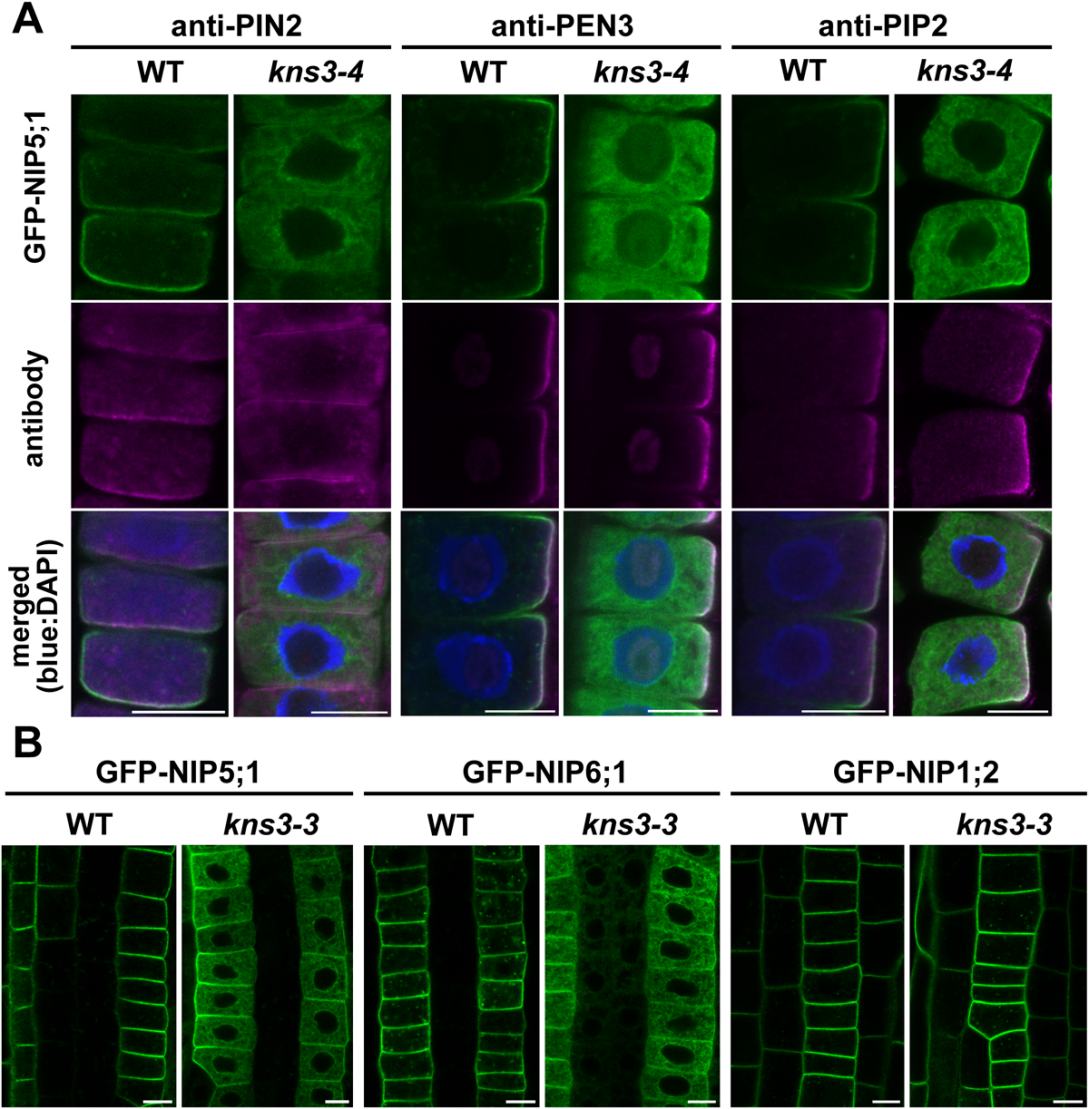
KNS3 functions in the trafficking of specific plasma membrane proteins, including NIP5;1 and NIP6;1. (A) Immunofluorescence of PIN2, PEN3, and PIP2 in the root epidermal cells of the wild-type (WT) and *kns3-4* mutant. GFP-NIP5;1 (green), endogenous PIN2, PEN3, and PIP2 detected by antibodies (magenta), and nuclei stained with 2 μg ml DAPI (blue) are shown. (B) GFP-NIP5;1, GFP-NIP6;1, and GFP-NIP1;2 expressed under the control of the NIP5;1 promoter in the WT and *kns3-3* background. Plants were grown on solid medium containing 30 μM B for 7–10 d. Scale bar=10 μm.

To examine whether KNS3 specifically affects the intracellular transport of NIP5;1, we investigated the localization of an auxin transporter, PIN2 (Abas *et al.*, 2006), an ATP-binding cassette transporter, PEN3/PLEIOTROPIC DRUG RESISTANCE8 (Langowski *et al.*, 2010), and an aquaporin, PIP2 (Maurel *et al.*, 2015), in *kns3-4* and the WT (Col-0). In the WT, immunofluorescence with anti-PIN2, anti-PEN3, and anti-PIP2 antibodies showed the co-localization of these proteins with GFP-NIP5;1 in the PM of epidermal cells. In *kns3-4* mutant cells, these three proteins were localized in the PM, whereas GFP-NIP5;1 accumulated in the ER (Fig. 3A). These results indicate that KNS3 is not required for the trafficking of PIN2, PEN3, and PIP2 to the PM.

We also examined the localization of NIP6;1 and NIP1;2, which belong to the NIP subfamily. To examine their localization in the root epidermis, we used plants expressing GFP-NIP6;1 and GFP-NIP1;2 under the control of the NIP5;1 promoter. In root epidermal cells, GFP-NIP6;1 was localized to the PM in WT but to the ER and PM in *kns3-3*, similar to GFP-NIP5;1. However, GFP-NIP1;2 was localized in the PM of both WT and *kns3-3* mutants (Fig. 3B). Therefore, ER retention in the *kns3* mutant was observed only for NIP5;1 and its close homolog NIP6;1 among the PM-localized proteins tested.

It has been reported that NIP5;1 is localized in the PM in a polar manner toward the soil side in epidermal cells and that polar localization is important for the efficient transport of B in roots (Wang *et al.*, 2017). Therefore, we investigated whether KNS3 functions in the polar trafficking of NIP5;1. In optical longitudinal sections at the center of the WT root, we observed GFP-NIP5;1 in the soil-side PM domain but not in the stele-side PM domain in the epidermal cells (Supplementary Fig. S2). Comparison with the signal of FM4-64, a lipophilic styryl dye that stains the PM, confirmed its polar localization (Supplementary Fig. S2). Although intracellular signals were observed, GFP-NIP5;1 in the PM of epidermal cells of *kns3-3* (Supplementary Fig. S2) showed a polar localization. These results indicate that KNS3 is involved in ER exit but not in polar trafficking to the PM.

### Defects in silique development but not pollen surface structure of *kns3* mutants are partially rescued by higher boron supply

**Fig. 4.**
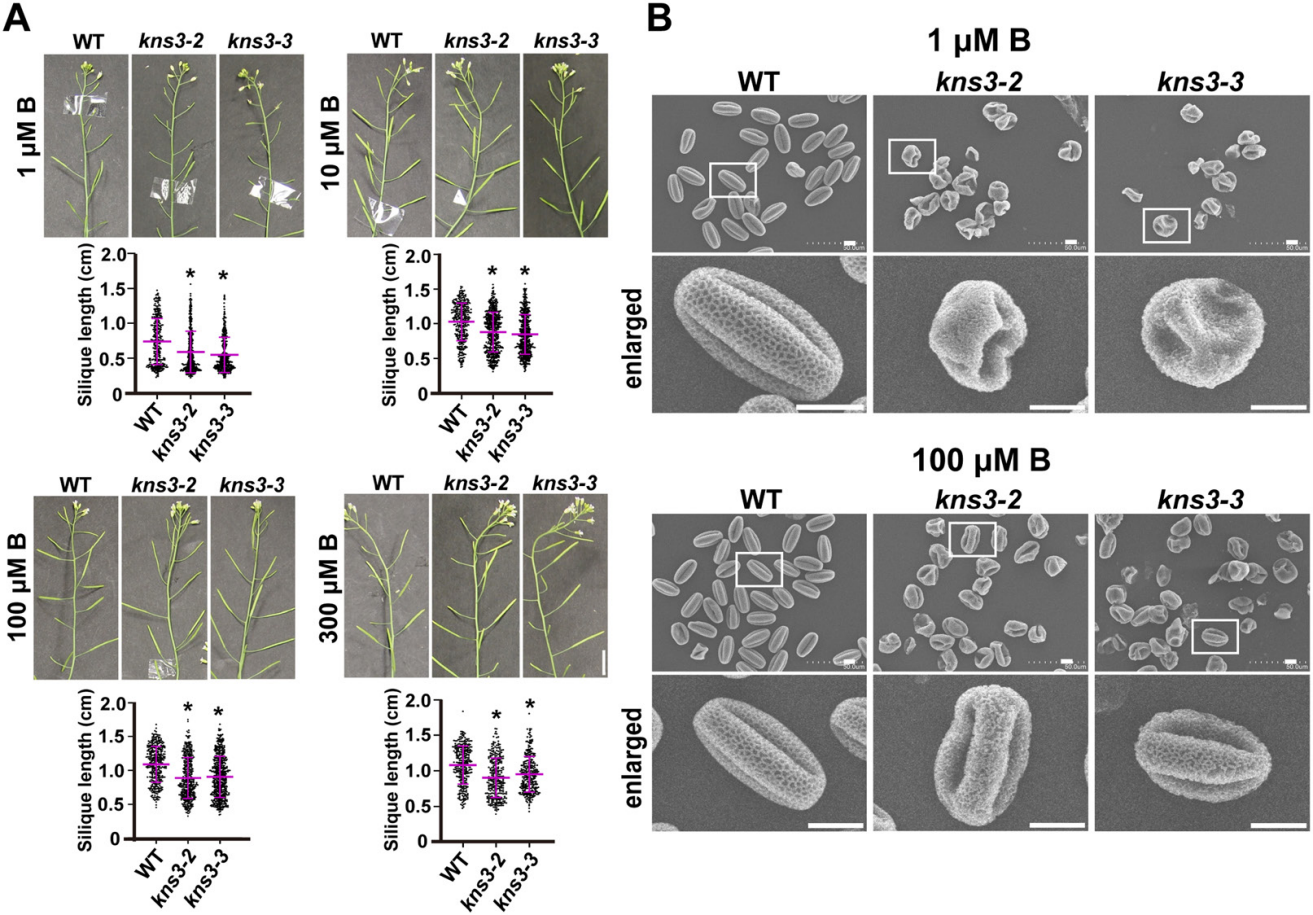
Defects in silique development but not pollen surface structure of *kns3* mutants were partially rescued by a high B supply. (A) Siliques of wild-type (WT), *kns3-2*, and *kns3-3* plants grown hydroponically with 30 µM B for 10–12 d and then with 1, 10, 100, or 300 μM B for 30 d. The lengths of 191–386 siliques from three plants were measured. Data represent the mean ±SD. Asterisks indicate mutants that showed significant differences from the WT (*P<0.01; Mann–Whitney U test). (B) Scanning electron micrographs of pollen grains from WT and *kns3* mutants grown hydroponically with 30 µM B for 10–12 d and then with 1 μM or 100 μM B for 20 d. More than 50 pollen grains were observed, and representative images are shown. Scale bars=1 cm (A) and 10 μm (B).

Given that GFP-NIP5;1 and GFP-NIP6;1 showed significant ER retention in the *kns3* mutants (Figs 2C, 3B), B transport was possibly affected in these mutants. To investigate the effect of B conditions on the phenotypes of the *kns3* mutants, *kns3-2*, *kns3-3*, *nip5;1-1*, a *NIP5;1* T-DNA insertion mutant (Takano *et al.*, 2006), and WT plants were grown on solid medium with low to sufficient (0.03, 0.3, 3, and 30 µM) B concentrations for 7 d. The *kns3* mutants did not show any significant differences from the WT in terms of shoot fresh weight or root length under different B conditions, whereas both values were significantly reduced in *nip5;1-1*, as reported previously (Supplementary Fig. S3A) (Takano *et al.*, 2006). Subsequently, *kns3-3*, *nip6;1-1*, a *NIP6;1* T-DNA insertion mutant (Tanaka *et al.*, 2008), and WT plants were grown in a hydroponic system with low (0.1 µM), moderately low (1 µM), and sufficient (100 µM) B concentrations. As previously reported, the *nip6;1-1* mutant showed reduced expansion of young rosette leaves and loss of apical dominance under low B conditions but normal growth under sufficient B conditions (Tanaka *et al.*, 2008) (Supplementary Fig. S3B, C). In contrast to the *nip6;1-1* mutant, the *kns3* mutants grew normally with no visible growth defects until flowering under these conditions (Supplementary Fig. S3B, C). However, the siliques of *kns3-2* and *kns3-3* mutants were shorter than those of the WT in low to moderately high B conditions: silique lengths of *kns3-2* were 80, 85, 82, and 84% of those of the WT in 1, 10, 100, and 300 μM B, respectively, and those of *kns3-3* were 75, 82, 83, and 88% of those of the WT, respectively (Fig. 4A). Although the siliques of *kns3* mutants were shorter than those of the WT under all conditions tested, the difference decreased as the B supply was increased. We then analyzed the pollen structure of *kns3-2* and *kns3-3* by scanning electron microscopy in plants grown with 1 µM and 100 µM B supply. Under both conditions, all pollen grains had a collapsed exine structure, in contrast to the reticulate pattern of the WT pollen surface (Fig. 4B).

To investigate the possible alteration of B transport in the *kns3* mutants, WT and *kns3* mutants were grown hydroponically with 30 μM B for 10–12 d and then transferred to low (1 µM) or sufficient (100 µM) B conditions for an additional 15 d. B concentrations in the roots, rosette leaves, and shoot apices of *kns3-2 and kns3-3* mutants were not significantly different from those of the WT under both low and sufficient B conditions (Supplementary Fig. S3D). It is likely that the boric acid channels remaining in the PM of the cells of the *kns3* mutants sufficiently supported the uptake and translocation of B. It is possible that B transport in flowers is affected and that factors other than B are also involved in the phenotypes of pollen and siliques in the *kns3* mutants.

### Two predicted homologs of KNS3 are also important for the endoplasmic reticulum exit of NIP5;1

**Fig. 5.**
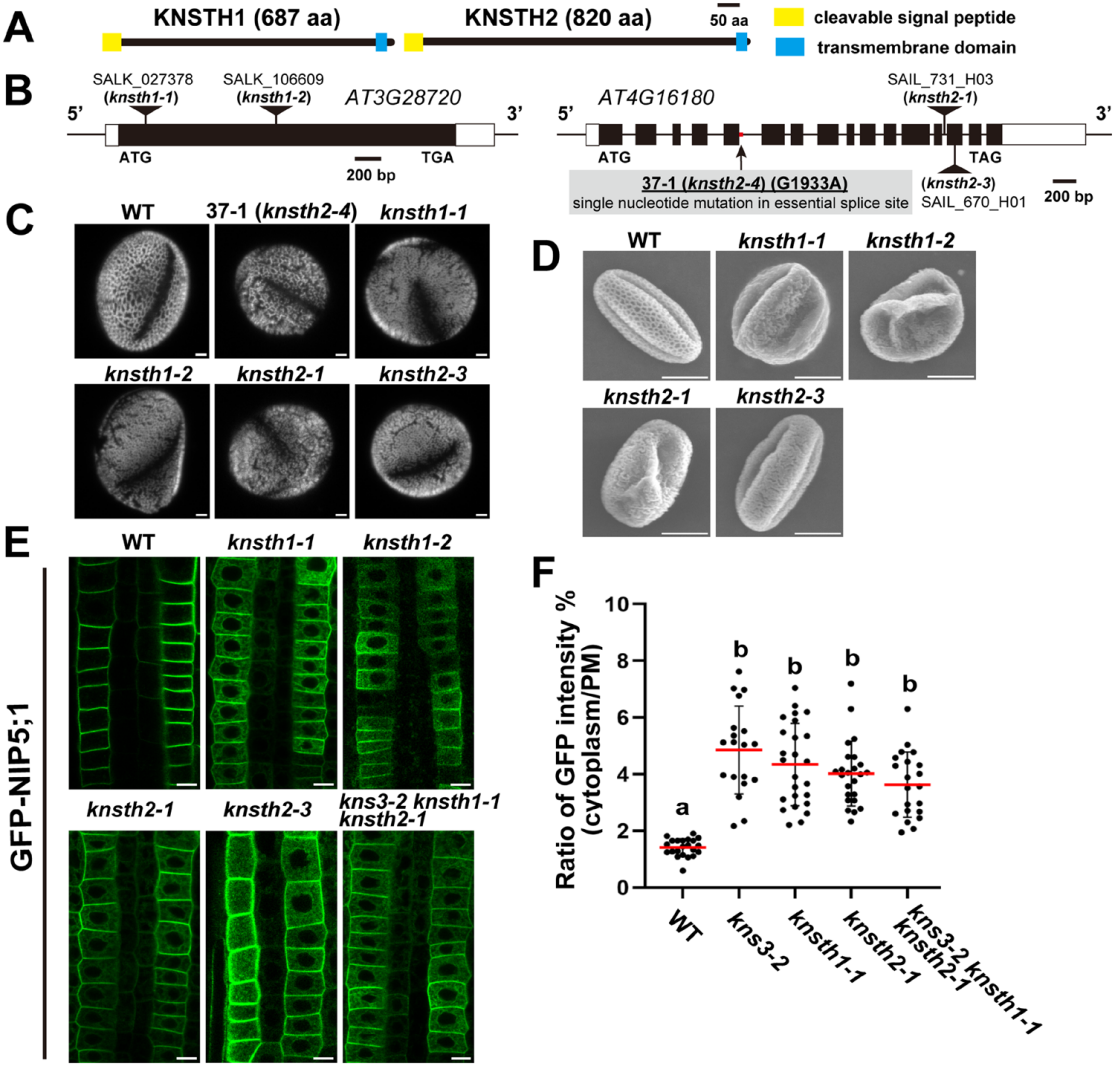
Two predicted homologs of KNS3 are also important for the endoplasmic reticulum exit of NIP5;1. (A) Topology of KNSTH1 and KNSTH2 containing an N-terminal signal peptide and a C-terminal transmembrane domain. (B) The exon–intron structures of KNSTH1 (At3G28720) and KNSTH2 (At4G16180). Positions for T-DNA insertions in *knsth1-1* and *knsth2-1*, and a point mutation in line 37–1 (*knsth2-4*), are shown. (C) Pollen of wild-type (WT) and the *knsth1* and *knsth2* mutants. Pollen grains were stained with 0.001% Auramine O. (D) SEM images of pollen grains from WT, *knsth1*, and *knsth2* mutants. Plants were grown in pots with vermiculite supplied with 1/1000 diluted Hyponex solution. (E) GFP-NIP5;1 in *kns3*, *knsth1*, and *knsth2* single T-DNA insertion mutants and the triple mutant. Plants were grown on solid medium containing 30 μM B for 7–10 d. (F) Ratio of GFP intensity in the cytoplasm and plasma membrane. Dot plots show the distribution among 23 (WT), 23 (*kns3-2*), 25 (*knsth1-1*), 24 (*knsth2-1*), and 25 (*kns3-2 knsth1-1 knsth2-1*) cells from three or four independent primary roots. Data represent the mean (red lines) ±SD (bars). Different letters indicate significant differences based on Tukey’s test (*P*<0.01). Scale bars=2.5 μm (C) and 10 μm (D, E).

To investigate the possible functional redundancy of KNS3, we searched for KNS3 homologs in the *A. thaliana* genome (TAIR10.0). A protein BLAST search in the National Center for Biotechnological Information (NCBI) database (https://blast.ncbi.nlm.nih.gov/Blast.cgi) showed that At3g28720.1 and At4g16180.2 have 26% and 28% identity with KNS3 (At5G58100.1), respectively. These two proteins were predicted to have an N-terminal signal peptide and a C-terminal transmembrane domain similar to those of KNS3 by the Philius program (Reynolds *et al.*, 2008) (Fig. 5A) and the AlphaFold protein structure database (Varadi *et al.*, 2022) (Supplementary Fig. S4B, C). Thus, we named At3g28720 and At4g16180 as KNS THREE HOMOLOGS (KNSTH) 1 and 2, respectively. Importantly, we found that mutant line 37–1 had a single-nucleotide mutation in the essential splice site (C/T) downstream of exon 5 in *KNSTH2* (Fig. 5B). We named line 37–1 as *knsth2-4*. We observed a defective pollen wall structure in the *knsth1-1* and *knsth2-1* T-DNA insertion lines, similar to that in *kns3* mutants (Fig. 5C, D). Moreover, the defective pollen wall was similarly observed in *kns3-2 knsth1-1*, *kns3-2 knsth2-1*, and *knsth1-1 knsth2-1* double mutants, and in the *kns3-2 knsth1-1 knsth2-1* triple mutant (Supplementary Fig. S5) We then introduced the GFP-NIP5;1 construct into the *knsth1* and *knsth2* mutants and observed fluorescence in root epidermal cells. GFP-NIP5;1 was localized in the ER and PM in *knsth1-1*, *knsth1-2*, *knsth2-1*, *knsth2-3*, and the *kns3-2 knsth1-1 knsth2-1* triple mutant (Fig. 5E), as well as in *kns3* mutants (Fig. 2C). The cytoplasm/PM ratios of the GFP-NIP5;1 signal were significantly higher in the *kns3-2*, *knsth1-1*, *knsth1-2*, and triple mutant plants than in the WT plants and were not significantly different among these mutants (Fig. 5F). GFP-NIP5;1 in the PM showed polar localization in the *kns3-3* single mutant and the triple mutant, similar to that in the WT (Supplementary Fig. S2). These results suggest that KNS3 and its two homologs work together in the ER exit of NIP5;1 and pollen wall formation.

### KNS3 is localized in the endoplasmic reticulum and Golgi, whereas KNSTH2 is mainly localized in the endoplasmic reticulum

**Fig. 6.**
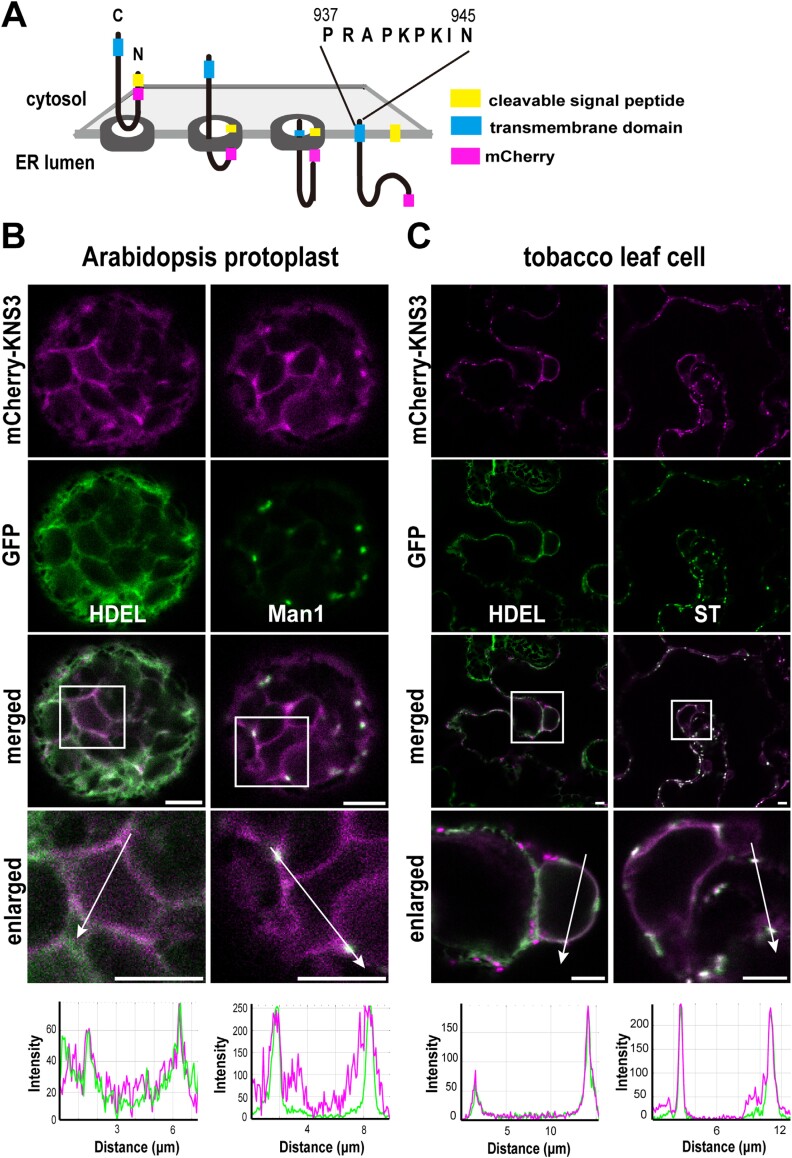
mCherry-KNS3 is localized in the endoplasmic reticulum and Golgi. (A) Topology of KNS3 with an N-terminal signal peptide and a transmembrane domain. In the mCherry-KNS3 construct, mCherry was inserted after the signal peptide. (B) mCherry-KNS3 and GFP-HDEL or Man1-GFP expressed in protoplasts from wild-type (Col-0) Arabidopsis leaf mesophyll cells using polyethylene glycol-mediated transformation. (C) mCherry-KNS3 and GFP-HDEL, or ST-YFP in *N. benthamiana* leaf epidermal cells after agroinfiltration. Scale bars=5 μm.

KNS3 was predicted to contain an N-terminal signal peptide and a C-terminal transmembrane domain (Fig. 2A; Supplementary Fig. S4A). It is probable that KNS3 is inserted into the ER membrane, its N-terminal soluble region is located in the ER lumen, and its short (nine amino acids) C-terminal tail is located in the cytosol. To investigate the intracellular localization of KNS3, we designed a *ProUBQ10:mCherry-KNS3* construct, in which the mCherry sequence was located downstream of the signal peptide sequence (Fig. 6A). We examined KNS3 localization in protoplasts from the leaf mesophyll cells of WT Arabidopsis by PEG-mediated transformation. In protoplasts, mCherry-KNS3 showed ring- and network-like localization that overlapped with the ER marker GFP-HDEL, and showed punctate structures that co-localized with the Golgi marker Man1-GFP (Nebenführ *et al.*, 1999) (Fig. 6B). We also introduced the *mCherry-KNS3* construct into tobacco leaves by agroinfiltration. In the epidermal cells, mCherry-KNS3 was observed in ring-like and punctate patterns. In the ring-like structures, mCherry-KNS3 co-localized with the ER marker GFP-HDEL (Nebenführ *et al.*, 2000) (Fig. 6C). In the punctate structures, mCherry-KNS3 co-localized with the *trans*-Golgi marker ST-YFP (Saint-Jore *et al.*, 2002) (Fig. 6C). Line plot analysis confirmed co-localization. In summary, mCherry-KNS3 was detected in the ER and Golgi apparatus in both Arabidopsis protoplasts and tobacco leaf cells.

**Fig. 7.**
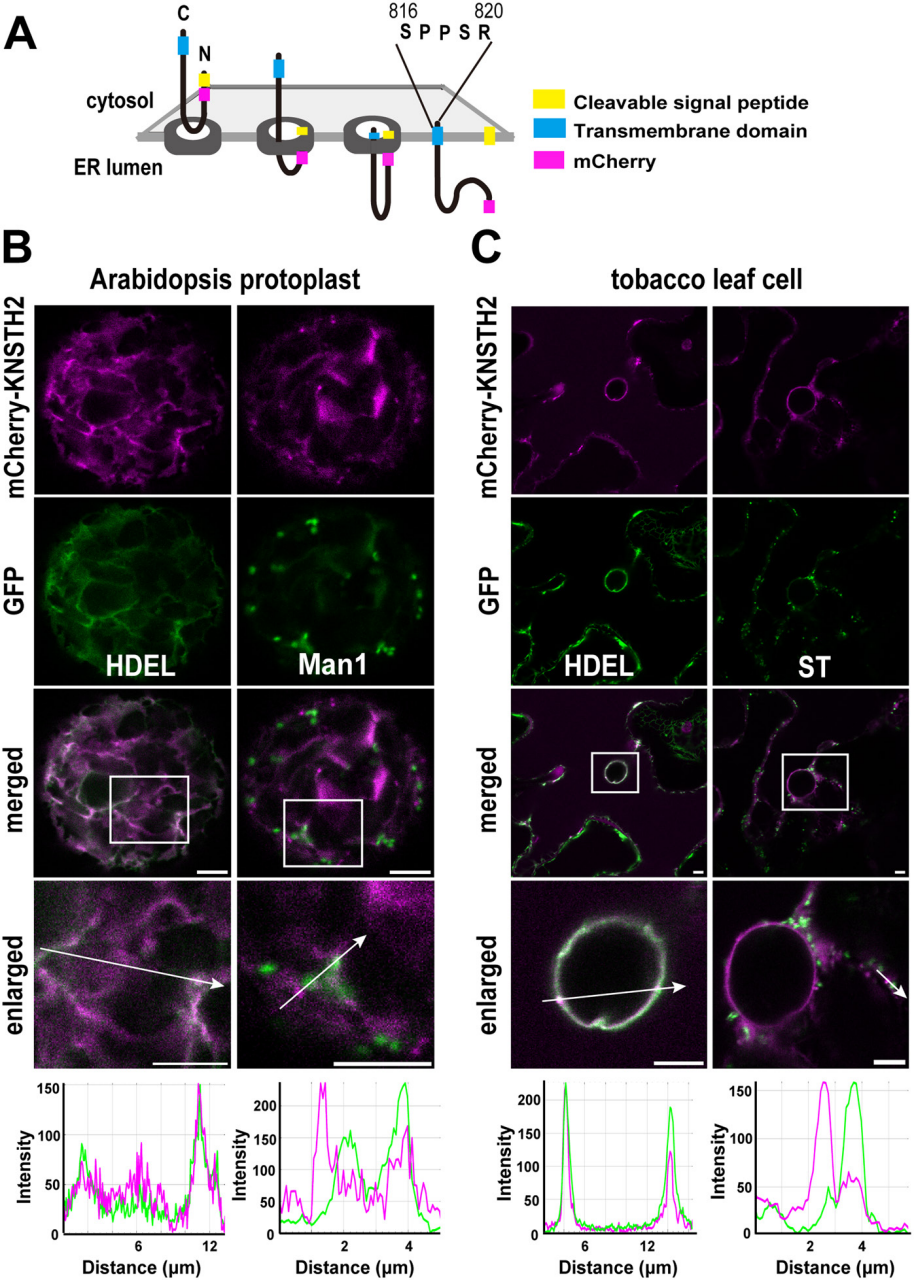
mCherry-KNSTH2 is mainly localized in the endoplasmic reticulum. (A) Topology of KNSTH2 with an N-terminal signal peptide and a transmembrane domain. In the mCherry-KNSTH2 construct, mCherry was inserted after the signal peptide. (B) mCherry-KNSTH2 and GFP-HDEL or Man1-GFP expressed in protoplasts from leaf mesophyll cells of wild-type (Col-0) Arabidopsis using polyethylene glycol-mediated transformation. (C) mCherry-KNSTH2 and GFP-HDEL or ST-YFP expressed in *N. benthamiana* leaf epidermal cells by agroinfiltration. Scale bars=5 μm.

Next, we designed a *ProUBQ10:mCherry-KNSTH2* construct, similar to the *mCherry-KNS3* construct (Fig. 7A). We examined KNSTH2 localization in protoplasts from leaf mesophyll cells of Arabidopsis WT plants. In protoplasts, mCherry-KNSTH2 showed ring-like, network-like, and occasionally punctate localization. In the ring- and network-like structures, mCherry-KNSTH2 overlapped with GFP-HDEL (Fig. 7B). mCherry-KNSTH2 did not significantly overlap with Man1-GFP (Fig. 7B). In tobacco leaf epidermal cells, mCherry-KNSTH2 exhibited ring-like and occasional punctate structures. In the ring-like structures, mCherry-KNSTH2 overlapped with GFP-HDEL. mCherry-KNSTH2 did not co-localize with ST-YFP (Fig. 7C). In summary, mCherry-KNSTH2 was observed mainly in the ER of Arabidopsis protoplasts and tobacco leaf cells.

We also designed a *ProUBQ10:mCherry-KNSTH1* construct, similar to mCherry-KNS3 and mCherry-KNSTH2. However, the introduction of this construct into competent cells of various *Escherichia coli* strains resulted in the rare appearance of colonies. Plasmids from these rare colonies contained mutations that caused amino acid substitutions or deletions in KNSTH1. Therefore, the toxicity of KNSTH1 hampered analysis of its localization. We also introduced *mCherry-KNSTH1* PCR products into the protoplasts of Arabidopsis leaf mesophyll cells (Lu *et al.*, 2013). Although a control experiment using *mCherry-HDEL* PCR products showed mCherry signals, we failed to detect any fluorescence from mCherry-KNSTH1.

### K941, K943, and I944 in the C-terminal tail of KNS3 are important for trafficking from the Golgi to the endoplasmic reticulum

**Fig. 8.**
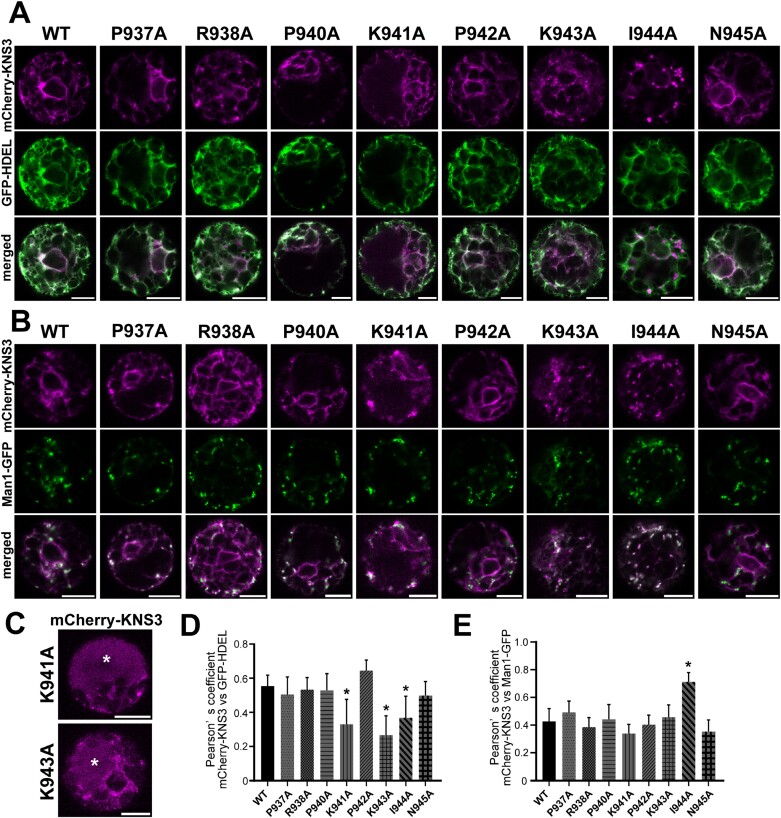
K941, K943, and I944 in the C-terminal tail of KNS3 are important for trafficking from the Golgi to the endoplasmic reticulum. (A, B) mCherry-KNS3 wild type (WT) and variants expressed with GFP-HDEL (A) or Man1-GFP (B) in protoplasts of WT (Col-0) Arabidopsis leaf mesophyll cells. (C) mCherry-KNS3 K941A and K943A were expressed in protoplasts of WT (Col-0) Arabidopsis leaf mesophyll cells. Asterisks indicate the vacuole. Scale bars=10 μm. (D, E) Pearson correlation coefficients of mCherry-KNS3 WT and variants with GFP-HDEL (D) or Man1-GFP (E). Data represent the mean ±SD of 10–16 protoplasts. Asterisks denote significant differences between the WT and variants (*P<0.01; Dunnett’s test).

mCherry-KNS3 was localized in the ER and Golgi apparatus in both Arabidopsis protoplasts and tobacco leaf cells (Fig. 6). To explore the importance of amino acids in KNS3 for trafficking between the ER and Golgi apparatus, we performed alanine scanning mutagenesis of the cytosolic C-terminal tail (PRAPKPKIN). We expressed mCherry-KNS3 WT and variants with the ER marker GFP-HDEL or the Golgi marker Man1-GFP in protoplasts from Arabidopsis leaf mesophyll cells. mCherry-KNS3 WT, P937A, R938A, P940A, P942A, and N945A were localized in ring- and network-like structures co-labeled with the ER marker and in punctate structures co-labeled with the Golgi marker (Fig. 8A, B). mCherry-KNS3 K941A and K943A were also localized in ring- and network-like structures and punctate structures (Fig. 8A, B), whereas they occasionally showed diffuse mCherry signals in the cells (four of 20 protoplasts with K941A and four of 21 protoplasts with K943A) (Fig. 8C). Considering that leaf mesophyll protoplasts are largely occupied by vacuoles (for examples, refer to images of tonoplast or vacuolar labeling in Aluri and Büttner, 2007; Kang *et al.*, 2012; Lee *et al.*, 2013), we judged that the diffuse mCherry signals were in the vacuole. mCherry-KNS3 I944A was not observed in ring- or network-like structures but was mainly in punctate structures co-labeled with Man1-GFP (Fig. 8A, B). Pearson correlation coefficients between mCherry-KNS3 P937A, R938A, P940A, P942A, N945A, and GFP-HDEL (0.50–0.65) were similar to that of mCherry-KNS3 WT (0.56), while those of K941A, K943A, and I944A (0.27–0.37) were significantly lower (Fig. 8D). Pearson correlation coefficients between mCherry-KNS3 P937A, R938A, P940A, K941A, P942A, K943A, N945A and Man1-GFP (0.34–0.49) were similar to that of mCherry-KNS3 WT (0.43), while that of I944A (0.71) was significantly higher than that of the WT (Fig. 8E). We also expressed mCherry-KNS3 WT and variants with the ER marker GFP-HDEL or the Golgi marker ST-YFP in tobacco leaf epidermal cells. mCherry-KNS3 WT, P937A, R938A, P940A, P942A, and N945A were localized in the ER and Golgi apparatus (Supplementary Fig. S6A, B). Signals from mCherry-KNS3 K941A, K943A, and I944A were observed as punctate structures co-labeled with the Golgi marker (Supplementary Fig. S6B) and as diffuse signals (Supplementary Fig. S6A, B). Since leaf epidermal cells are largely occupied by vacuoles (Foresti *et al.*, 2010; Scabone *et al.*, 2011; Vieira *et al.*, 2019; Wang *et al.*, 2020), the diffuse mCherry signals were considered to be in the vacuole. The results are summarized in Supplementary Table S3. These results suggest that K941, K943, and I944 are important for the retrograde trafficking of KNS3 from the Golgi to the ER, and that the alanine-substituted variants were mistargeted to the vacuole from the Golgi.

### Mutations in the C-terminal tail do not affect the endoplasmic reticulum localization of KNSTH2

**Fig. 9.**
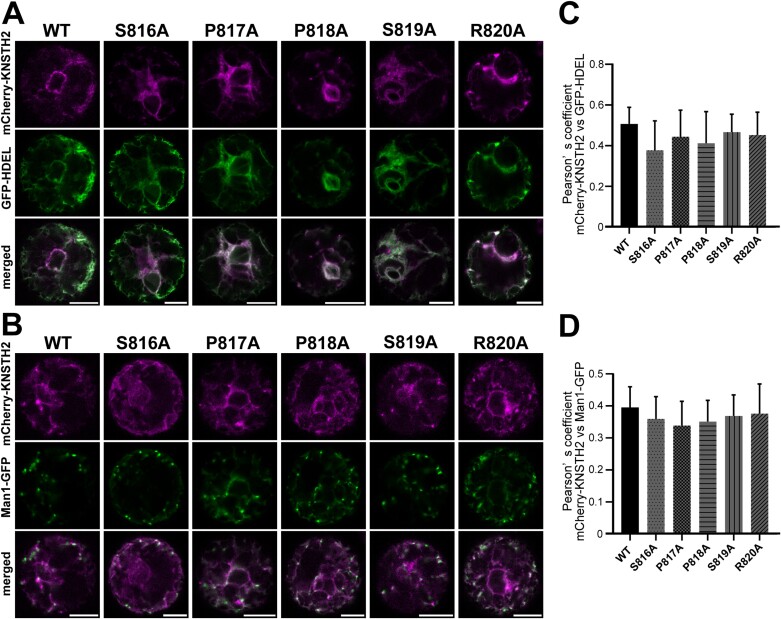
Mutations in the C-terminal tail do not affect the endoplasmic reticulum localization of KNSTH2. (A, B) mCherry-KNSTH2 WT and variants expressed with GFP-HDEL (A) or Man1-GFP (B) in protoplasts extracted from wild-type (WT) (Col-0) Arabidopsis leaf mesophyll cells. (C, D) Pearson coefficients of mCherry-KNSTH2 WT and variants with GFP-HDEL (C) or Man 1-GFP (D). Data represent the mean ±SD of 10 or 11 protoplasts. No significant differences were observed (*P*>0.05; Dunnett’s test). Scale bar=10 μm.

KNSTH2 was mainly localized in the ER in both Arabidopsis protoplasts and tobacco leaf cells (Fig. 7). To investigate the possible shuttling of KNSTH2 between the ER and Golgi and the involvement of its C-terminal tail in trafficking, we performed alanine scanning mutagenesis of the cytosolic C-terminal tail (SPPSR) of KNSTH2 (Fig. 7A). In Arabidopsis protoplasts, mCherry-KNSTH2 WT, S816A, P817A, P818A, S819A, and R820A showed ring- and network-like structures co-labeled with GFP-HDEL (Fig. 9A). These five variants and the WT hardly showed punctate localization and rarely co-localized with Man1-GFP (Fig. 9B). Pearson correlation coefficients between mCherry-KNSTH2 variants and GFP-HDEL (0.41–0.44) were similar to that of mCherry-KNSTH2 WT and GFP-HDEL (0.51) (Fig. 9C). Pearson correlation coefficients between mCherry-KNSTH2 variants and Man1-GFP (0.32–0.36) were similar to that of mCherry-KNSTH2 WT and Man1-GFP (0.39) (Fig. 9D). We also expressed mCherry-KNSTH2 WT and variants with GFP-HDEL or ST-YFP in tobacco leaf epidermal cells. Similar to mCherry-KNSTH2 WT, the variants co-localized well with the ER marker GFP-HDEL but not with the Golgi marker ST-YFP (Supplementary Fig. S7). In summary, mutations in the KNSTH2 C-terminal tail did not influence the ER localization of mCherry-KNSTH2 (Supplementary Table S3).

### KNSTH1 and KNSTH2 are important for the trafficking of KNS3 from the endoplasmic reticulum to the Golgi

To investigate whether KNS3 and its homologs affect the trafficking of each other, we expressed mCherry-KNS3 in protoplasts from the *knsth1* and *knsth2* mutants. In *knsth1-1* or *knsth2-1* cells, mCherry-KNS3 showed ring- and network-like structures and occasionally showed punctate structures, similar to those in the WT cells (Supplementary Fig. S8A, B). The Pearson correlation coefficients between mCherry-KNS3 and GFP-HDEL or Man1-GFP in *knsth1-1*, *knsth2-1*, and WT cells were not significantly different (Supplementary Fig. S8C, D). Subsequently, we expressed mCherry-KNSTH2 in protoplasts from the WT, *kns3-3*, and *knsth1-1*. mCherry-KNSTH2 showed ring- and network-like structures in *kns3-3* and *knsth1-1*, similar to those in the WT (Supplementary Fig. S8E, F). The Pearson correlation coefficients between mCherry-KNSTH2 and GFP-HDEL or Man1-GFP in the WT, *kns3-3*, and *knsth1-1* were not significantly different (Supplementary Fig. S8G, H). These results suggest that most KNS3 and KNSTH2 are localized in the ER, and the lack of their homologs does not affect steady-state localization.

**Fig. 10.**
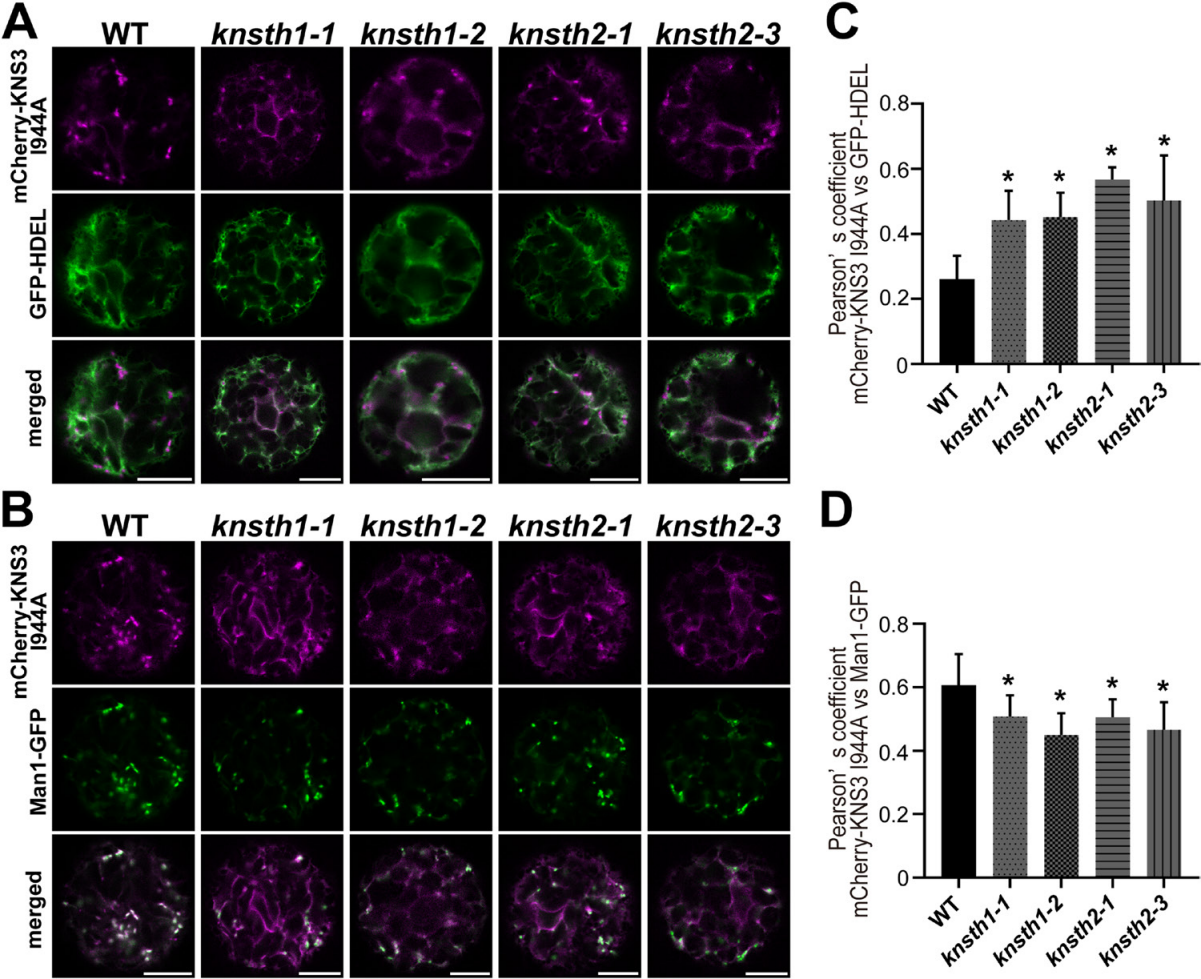
KNSTH1 and KNSTH2 are important for the trafficking of KNS3 from the endoplasmic reticulum to the Golgi. (A, B) mCherry-KNS3 I944A and GFP-HDEL (A) or Man1-GFP (B) in protoplasts from wild-type (WT) (Col-0), *knsth1-1*, *knsth1-2*, *knsth2-1*, and *knsth2-3* Arabidopsis leaf mesophyll cells. Scale bars=10 μm. (C, D) Pearson correlation coefficients of mCherry-KNS3 I944A with GFP-HDEL (C) or Man1-GFP (D). Data represent the mean ±SD of 10–17 protoplasts. Asterisks denote significant differences between the WT and the mutant lines (*P<0.01; Dunnett’s test).

We then analyzed the localization of mCherry-KNS3 I944A, for which we showed an increased co-localization with the Golgi apparatus in Arabidopsis leaf mesophyll protoplasts (Fig. 8B, E). Owing to the defect in the mCherry-KNS3 I944A variant in retrograde trafficking from the Golgi to the ER, we were able to analyze the contributions of KNSTH1 and KNSTH2 in the ER-to-Golgi trafficking of KNS3. In contrast to the punctate localization seen in WT protoplasts, mCherry-KNS3 I944A showed both ER-like and punctate localization in *knsth1-1*, *knsth1-2*, *knsth2-1*, and *knsth2-3* cells (Fig. 10A, B). The Pearson correlation coefficients between mCherry-KNS3 I944A and GFP-HDEL in the WT were approximately 0.3, whereas they were significantly higher, up to approximately 0.6, in *knsth1-1*, *knsth1-2*, *knsth2-1*, or *knsth2-3* cells. (Fig. 10C). The Pearson correlation coefficients between mCherry-KNS3 I944A and Man1-GFP in the WT were approximately 0.6, whereas they were significantly lower, at approximately 0.5, in *knsth1-1*, *knsth1-2*, *knsth2-1*, or *knsth2-3* cells (Fig. 10D). The results are summarized in Supplementary Table S3. These results indicated that KNSTH1 and KNSTH2 are important for the trafficking of KNS3 from the ER to the Golgi.

### Accumulation of KNS3 and its two homologs is mutually dependent

**Fig. 11.**
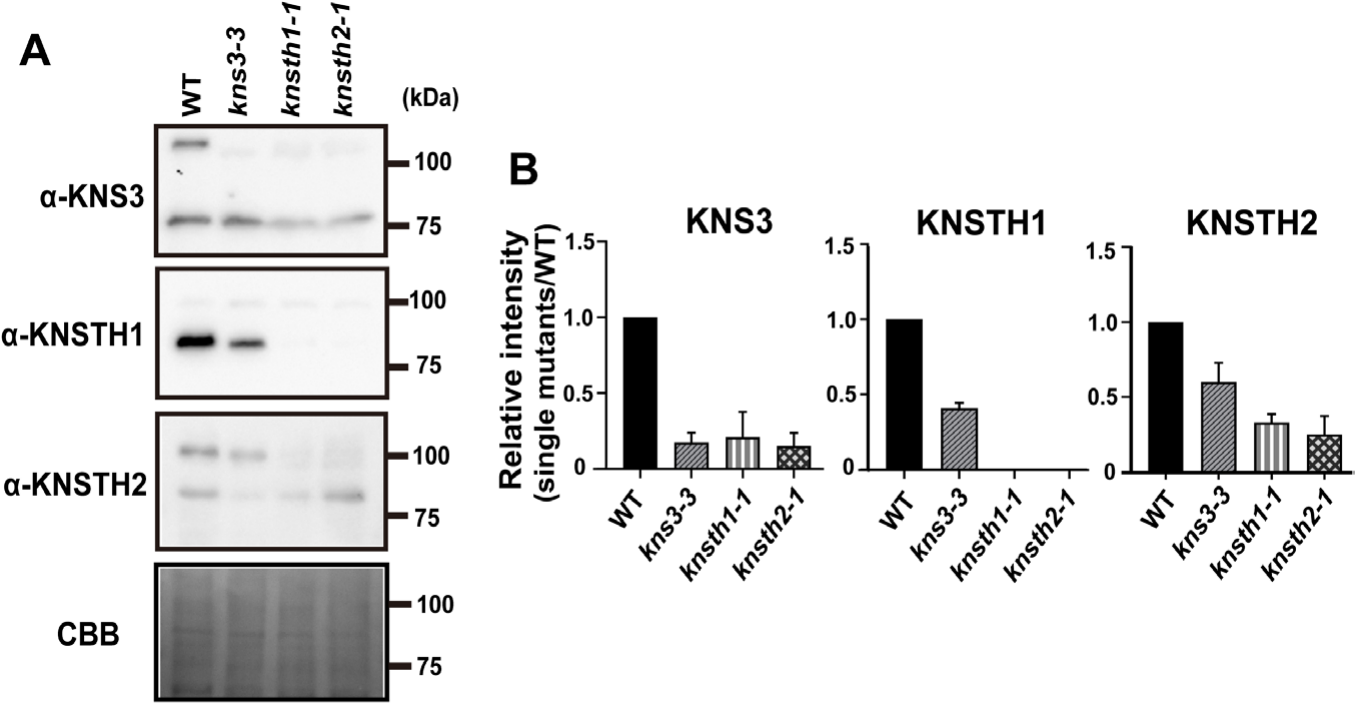
The accumulation of KNS3 and its two homologs is mutually dependent. (A) Immunoblotting of KNS3, KNSTH1, and KNSTH2 proteins in microsomal fractions. wild-type (WT), *kns3-3*, *knsth1-1*, and *knsth2-1* plants were grown on solid medium containing 30 μM B for 3 weeks. Approximately 60 plants were used as one sample for microsome preparation. Immunoblot analysis was performed using anti-KNS3, anti-KNSTH1, and anti-KNSTH2 antibodies. (B) Densitometric quantification. Data represent the mean ±SD of three independent experiments.

The rate of ER retention of GFP-NIP5;1 in the *kns3-2 knsth1-1 knsth2-1* triple mutant was not significantly different from that in the single mutants (Fig. 5F). This result suggests that KNS3 shares a function with its two homologs in the ER exit of NIP5;1. To investigate the relation between KNS3 and its two homologs at the protein accumulation level, we quantified the protein in the roots of WT, *kns3-3*, *knsth1-1*, and *knsth2-1* plants using newly generated anti-KNS3, KNSTH1, and KNSTH2 antibodies (Fig. 11A). The predicted molecular weights of KNS3, KNSTH1, and KNTH2 were 107.7, 76.5, and 92.4 kDa, respectively. In the microsome preparations, anti-KNS3 detected a clear band at approximately 120 kDa in the WT strain and a faint band at approximately 115 kDa in the *kns3-3* mutant (Fig. 11A). The slight accumulation of the smaller protein was possibly due to translation from an aberrant mRNA containing a part of the T-DNA sequence (Fig. 2A). Anti*-*KNSTH1 detected clear bands at approximately 90 kDa in the WT but not in the *knsth1-1* mutant. Anti-KNSTH2 detected clear bands at approximately 105 kDa in the WT and faint bands at approximately 105 kDa and 100 kDa in the *knsth2-1* mutant. This faint expression was possibly due to translation from an mRNA in which the 13th intron, including the T-DNA sequence, was spliced out (Fig. 5B). These results indicated that the antibodies specifically detected the respective proteins. We then analyzed protein accumulation in the homolog mutants. The band intensities of KNS3 were approximately 20% and 15% in *knsth1-1* and *knsth2-1*, respectively, compared with those in the WT (Fig. 11B). The band intensity of KNSTH1 was approximately 40% in *kns3-3* and not detected in *knsth2-1*. The band intensities of KNSTH2 were approximately 60% and 30% in *kns3-3* and *knsth1-1*, respectively (Fig. 11A, B). These results indicate that KNS3, KNSTH1, and KNSTH2 are unstable when one of the two homologs is not expressed.

## Discussion

### Endoplasmic reticulum exit of NIP5;1 requires KNS3, KNSTH1, and KNSTH2

In our fluorescence imaging-based genetic screening, we identified six mutant lines (lines 1–3, 5–6, 10–6, 14–3, 15–2, and 37–1) in which GFP-NIP5;1 showed ER and PM localization (Fig. 1A). Using genetic mapping and whole-genome sequencing, we identified *KNS3* as the gene responsible for the four allelic lines (line 1–3, 10–6, 14–3, and 15–2; Fig. 2A) and *KNSTH2* as the gene responsible for line 37–1 (Fig. 5B). Retention of GFP-NIP5;1 in the ER was consistently observed in *kns3* and *knsth2* T-DNA insertion mutants (Figs 2C, 5E). We did not identify *KNSTH1* from the forward-genetic study. However, protein BLAST showed that KNSTH1 has 26% identity with KNS3, and the topology of KNSTH1 was similar to that of KNS3 (Fig. 5A; Supplementary Fig. S4B). In *knsth1* T-DNA insertion mutants, GFP-NIP5;1 showed ER and PM localization similar to that of the *kns3* and *knsth2* mutants (Fig. 5E). These results established that *KNS3* and the newly described proteins *KNSTH1* and *KNSTH2* are required for the ER exit of NIP5;1. Phylogenetic analysis showed that these three proteins are well conserved in mosses, lycophytes, and angiosperms (Supplementary Table S4; Supplementary Fig. S9), suggesting conserved roles in plant physiology.

### The defect in boron transport partially explains the defective fertility of *kns3* mutants

The *kns3* mutants showed defects in the ER exit of the boric acid channels NIP5;1 and NIP6;1 (Figs 2C, 3B). However, the *kns3* mutants did not show similar B-deficient phenotypes to the *nip5;1* or *nip6;1* mutants (Supplementary Fig. S3), and the B concentrations in the roots, rosette leaves, and shoot apices of the mutants were not significantly different from those of the WT (Supplementary Fig. S3D). This was probably because a sufficient number of boric acid channels were still transported to the PM in the mutants. Additionally, we noticed that the siliques of *kns3* mutants were shorter than those of the WT in the low B condition and were partially rescued by a normal to sufficient B supply (Fig. 4A). These results suggest that defective B transport in floral organs affects plant fertility. We also found that the pollen of *kns3* mutants showed an abnormal exine structure, irrespective of B conditions (Fig. 4B). It is likely that KNS3 is involved in the ER exit of boric acid channels and other cargoes responsible for the construction of the exine structure. It was previously demonstrated that the loss of function of the COPII components SEC31B, SEC23A and D, and SAR1B and C resulted in defective exine structures on the pollen surface and male-sterile phenotypes in Arabidopsis (Zhao *et al.*, 2016; Aboulela *et al.*, 2018; Liang *et al.*, 2020). These examples suggest that efficient ER exit is particularly important in tapetum cells, where massive secretion is required for pollen development.

### KNS3 and its homologs function as a possible cargo-receptor complex for boric acid channels

KNS3, KNSTH1, and KNSTH2 are predicted to contain a short cytosolic C-terminal tail, a single transmembrane domain, and a large luminal ER region (Figs 2A, 5A; Supplementary Fig. S4). These structures are similar to those of some ER-Golgi cargo receptors, such as ER-Golgi intermediate compartment-53 (ERGIC-53)/lectin mannose-binding 1 (LMAN1) in mammals and yeast, and p24 proteins in mammals, yeast, and plants (Zhang *et al.*, 2009; Pastor-Cantizano *et al.*, 2016). Generally, cargo receptors help package cargo proteins into vesicles for subsequent trafficking. ERGIC-53/LMAN1 and p24 proteins transport glycoproteins and glycosylphosphatidylinositol-anchored proteins, respectively, from the ER to the Golgi apparatus (Zhang *et al.*, 2009; Pastor-Cantizano *et al.*, 2016). Based on the similarity in the protein structure and function of cargo proteins at the ER exit, we speculate that KNS3, KNSTH1, and KNSHT2 are cargo receptors for the ER exit of boric acid channels and other cargoes.

We observed that the rate of ER retention of GFP-NIP5;1 in the *kns3-2 knsth1-1 knsth2-1* triple mutant was not significantly different from that in the single mutants (Fig. 5F). We also observed that the accumulation of KNS3, KNSTH1, and KNSTH2 was interdependent (Fig. 11B). Based on the ePlant database (Waese *et al.*, 2017), *KNS3*, *KNSTH1*, and *KNSTH2* are expressed ubiquitously in various tissues, but not in mature pollen (Honys and Twell, 2004; Nakabayashi *et al.*, 2005; Schmid *et al.*, 2005) (Supplementary Figs S10, S11, S12). These proteins likely function together in various cell types. Human ERGIC-53/LMAN1 forms a complex with MCFD2 (Zhang *et al.*, 2003, 2005). *Saccharomyces cerevisiae* p24 proteins, including Erp1p, Erp2p, Emp24p, and Erv25p, form a heteromeric complex, and their accumulation is interdependent (Marzioch *et al.*, 1999). Based on the analogy between these complexes, we hypothesize that KNS3 and its homologs function as a cargo-receptor complex. However, our co-immunoprecipitation assay using transgenic Arabidopsis expressing GFP-NIP5;1 and anti-KNS3/KNSTH1/KNSTH2 antibodies, and tobacco leaves expressing mCherry-KNS3 and GFP-NIP5;1 failed to detect interactions. This is probably because most of the GFP-NIP5;1 was localized in the PM, not in the ER. It will be important to test the direct interaction between the KNS3 complex and boric acid channels in the ER in future studies.

Next, we discuss the different roles of KNS3, KNSTH1, and KNSTH2 in the cargo-receptor complex. In Arabidopsis mesophyll protoplasts and tobacco leaf cells, mCherry-KNS3 localized to the ER and Golgi apparatus (Fig. 6).In contrast, the K941A and K943A variants localized to the Golgi and showed reduced ER localization (Fig. 8A, B, D), and the I944A variant localized to the Golgi but not to ring- and network-like structures in Arabidopsis protoplasts (Fig. 8A, B, E). The mCherry fluorescence derived from the K941A and K943A variants in Arabidopsis protoplasts and tobacco leaf cells and the I944A variant in tobacco leaf cells was also observed in the vacuoles (Fig. 8C; Supplementary Fig. S6A, B). These results suggest that the KxKI motif is important for Golgi-to-ER trafficking, and that defects in retrograde trafficking cause mistargeting of mCherry-KNS3 variants from the Golgi to the vacuole. In *S. cerevisiae*, the double lysine motif (KK) in the C-terminus of ERGIC-53/LMAN1, and the KxK motif in the C-terminal tail (-GKFFVKQKIL) of Erp1p, a member of the p24α subfamily, are important for retrograde trafficking from the Golgi to the ER (Schindler *et al*., 1993; Marzioch *et al.*, 1999; Pastor-Cantizano *et al.*, 2016). Based on this analogy, the cytosolic C-terminal KxKI motif in KNS3 may bind to the COPI complex for retrograde trafficking of KNS3 from the Golgi to the ER. This KxKI motif is conserved in KNS3 clade proteins from mosses to angiosperms (Supplementary Fig. S13). According to our working hypothesis, KNS3 is likely responsible for retrograde trafficking in the KNS3-KNSTH1-KNSTH2 complex (Supplementary Fig. S14).

KNSTH1 has a longer C-terminal tail (KRDRLFRNKRKQF) than that of KNS3 (PRAPKPKIN) or KNSTH2 (SPPSR). Previously, the double phenylalanine motif (FF) at the C-terminus of ERGIC-53/LMAN1 was shown to interact with the SEC23-SEC24 subcomplex of the COPII complex and to act as an ER-exit motif (Kappeler *et al.*, 1997). The C-terminal tail of the p24γ subfamily contains a ΦF motif (where Φ represents a bulky hydrophobic residue), which presumably allows binding to a COPII component for ER exit (Pastor-Cantizano *et al.*, 2016). These examples imply the possibility that KNSTH1 could bind to a COPII component with the ΦF motif in its C-terminal tail. The ΦF motif is conserved in the KNSTH1 clade proteins from mosses to angiosperms (Supplementary Fig. S13). Although we were unable to test this hypothesis due to the toxic effects of mCherry-KNSTH1 expression in bacterial and plant cells, KNSTH1 may be responsible for ER exit by binding to the COPII component in the KNS3-KNSTH1-KNSTH2–cargo complex (Supplementary Fig. S14).

In Arabidopsis mesophyll protoplasts and tobacco leaf cells, mCherry-KNSTH2 is localized mainly in the ER. Although we did not observe any changes in the localization of mCherry-KNSTH2 in the ER upon substitution of amino acids in the C-terminal tail (Fig. 9; Supplementary Fig. S7), we cannot rule out the possibility that KNSTH2 moves between the ER and Golgi, along with KNS3 and KNSTH1.

In conclusion, we revealed that *KNS3*, *KNSTH1*, and *KNSTH2* are important for the ER exit of the boric acid channel NIP5;1 and possibly other proteins involved in the construction of the pollen wall structure. We propose that KNS3, KNSTH1, and KNSTH2 form a cargo-receptor complex that interacts with boric acid channels and other cargoes in the ER, transporting them to the Golgi by packaging them into COPII vesicles (Supplementary Fig. S14). In the Golgi apparatus, the cargo receptor complex separates from the cargo and returns to the ER via COPI vesicles. Subsequently, the boric acid channels and other cargo molecules move from the *trans*-Golgi network to the PM via the secretion pathway.

## Supplementary data

The following supplementary data are available at *JXB* online.

Fig. S1. *At5g58100* (*KNS3*) is the causative gene for the ER retention of NIP5;1.

Fig. S2. GFP-NIP5;1 shows polar localization in mutants of *KNS3* and its homologs.

Fig. S3. Growth of *kns3* mutants under different B conditions.

Fig. S4. Topologies of KNS3, KNSTH1, and KNSTH2.

Fig. S5. Pollen structure in *kns3*, *knsth1*, and *knsth2* multiple mutants.

Fig. S6. K941, K943, and I944 in the C-terminal tail of KNS3 are important for its trafficking from the Golgi to the ER.

Fig. S7. Mutations in the C-terminal tail of KNSTH2 do not affect its ER localization.

Fig. S8. Localization of mCherry-KNS3 and KNSTH2 is unchanged in single mutants of their homologs.

Fig. S9. Phylogenetic tree of KNS3 and its homologs.

Fig. S10. Expression pattern of *KNS3*.

Fig. S11. Expression pattern of *KNSTH1*.

Fig. S12. Expression pattern of *KNSTH2*.

Fig. S13. Multiple alignments of the amino acid sequences of the C-terminal tail of KNS3 and its homologs.

Fig. S14. A working hypothesis of the functions of KNS3, KNSTH1, and KNSTH2 in an ER-Golgi cargo-receptor complex.

Table S1. SSLP markers for rough mapping.

Table S2. Primers used in this research.

Table S3. Summary of the localization of mCherry-KNS3 and mCherry-KNSTH2 in Arabidopsis protoplasts and tobacco leaf cells.

Table S4. List of proteins collected using BLAST in the NCBI database.

erae380_suppl_Supplementary_Figures_S1-S14-Tables_S1-S4

## Data Availability

All data supporting the findings of this study are available within the paper and within its supplementary data published online.
