# Supplementary material for: Arabidopsis KNS3 and its two homologs mediate endoplasmic reticulum-to-plasma membrane traffic of boric acid channels
Source: J Exp Bot. 2024 Oct 30;75(22):7046–65. doi: 10.1093/jxb/erae380 (PMC11629988; doi:10.1093/jxb/erae380)
Supplement: erae380_suppl_Supplementary_Figures_S1-S14-Tables_S1-S4 [file erae380_suppl_supplementary_figures_s1-s14-tables_s1-s4.pdf]

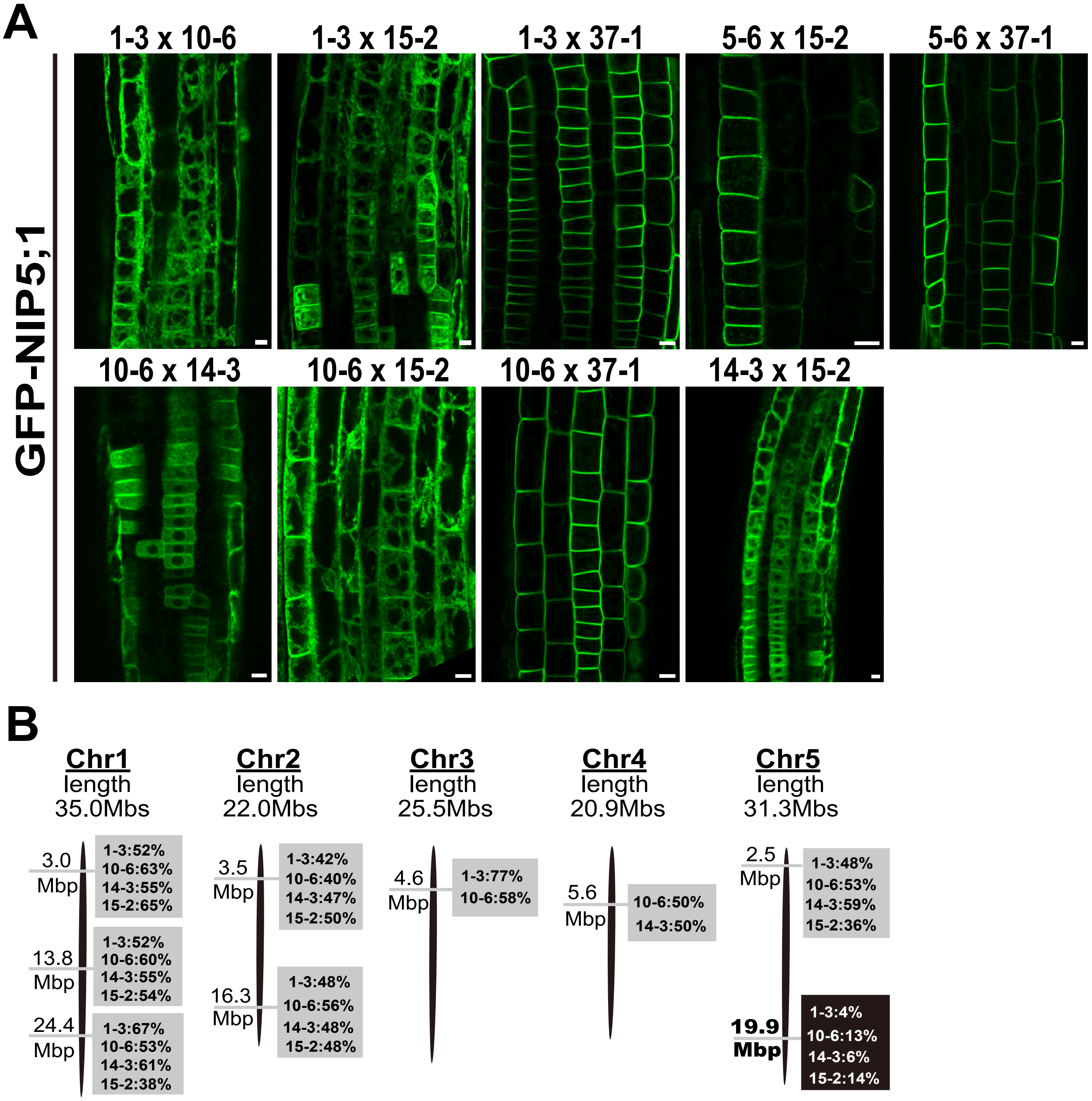

Fig. S1. *At5g58100* (*KNS3*) is the causative gene for the ER retention of NIP5;1.

(A) GFP-NIP5;1 in root epidermal cells of F1 plants between EMS mutants. Plants were grown on solid medium containing 30 μM B for 7–10 days. Scale bar=10 μm. (B) Summary of rough mapping using the F2 generation between ecotype *Ler* and line1–3, line10–6, line14–3, and line15–2. Recombination rates (%) at each marker position are indicated.

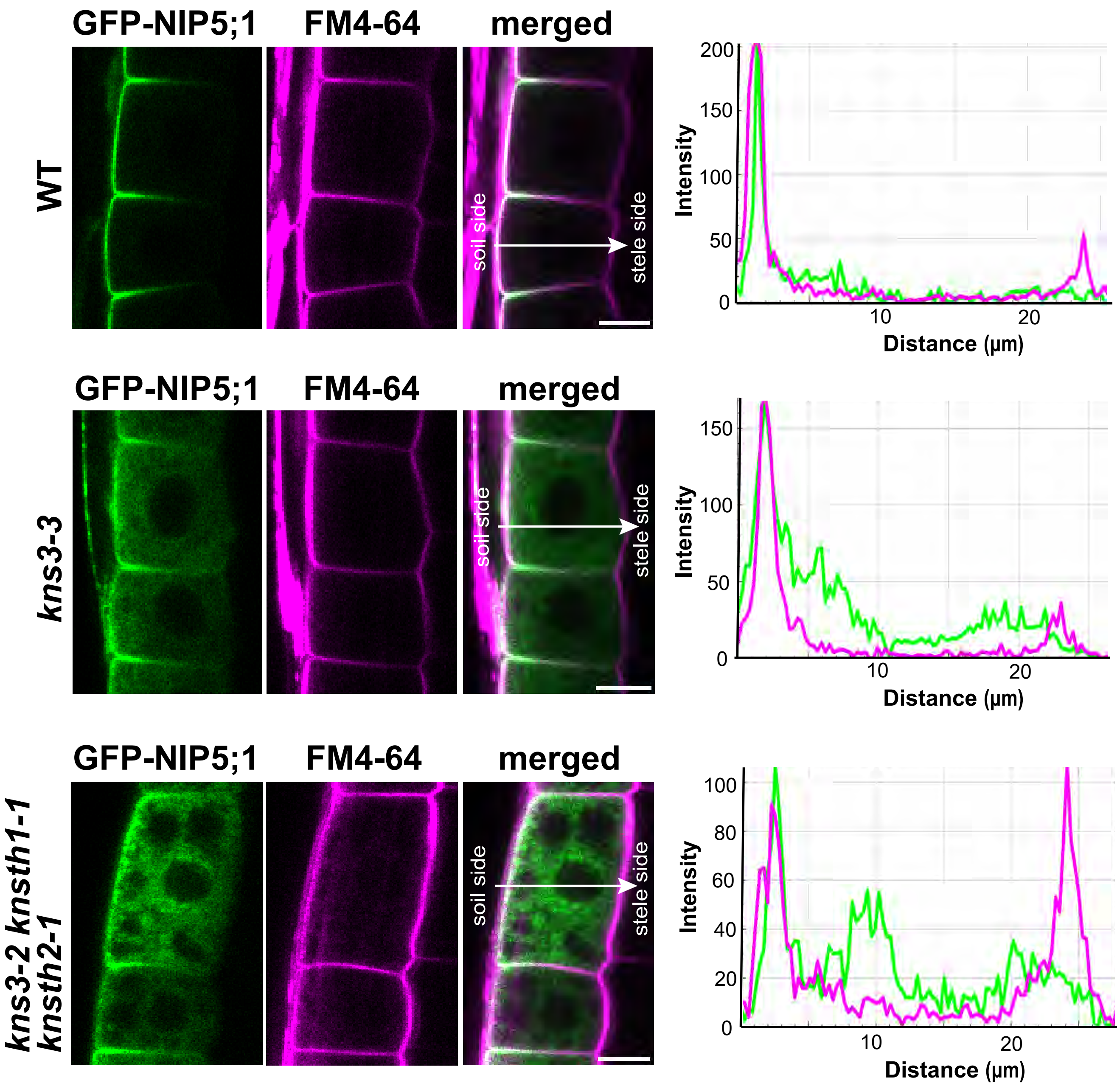

Fig. S2. GFP-NIP5;1 shows polar localization in mutants of *KNS3* and its homologs.

Polar localization of GFP-NIP5;1 in root epidermal cells of *nip5;1-1* (WT), a *KNS3* single mutant (*kns3-3*), and a triple mutant (*kns3-2 knsth1-1 knsth2-1*). Plant roots were stained with FM4-64 for 1 min and an optical longitudinal section of the root meristem zone was imaged by a confocal microscope. Plants were grown with 30 µM B for 4–7 days. Scale bar=10 µm.

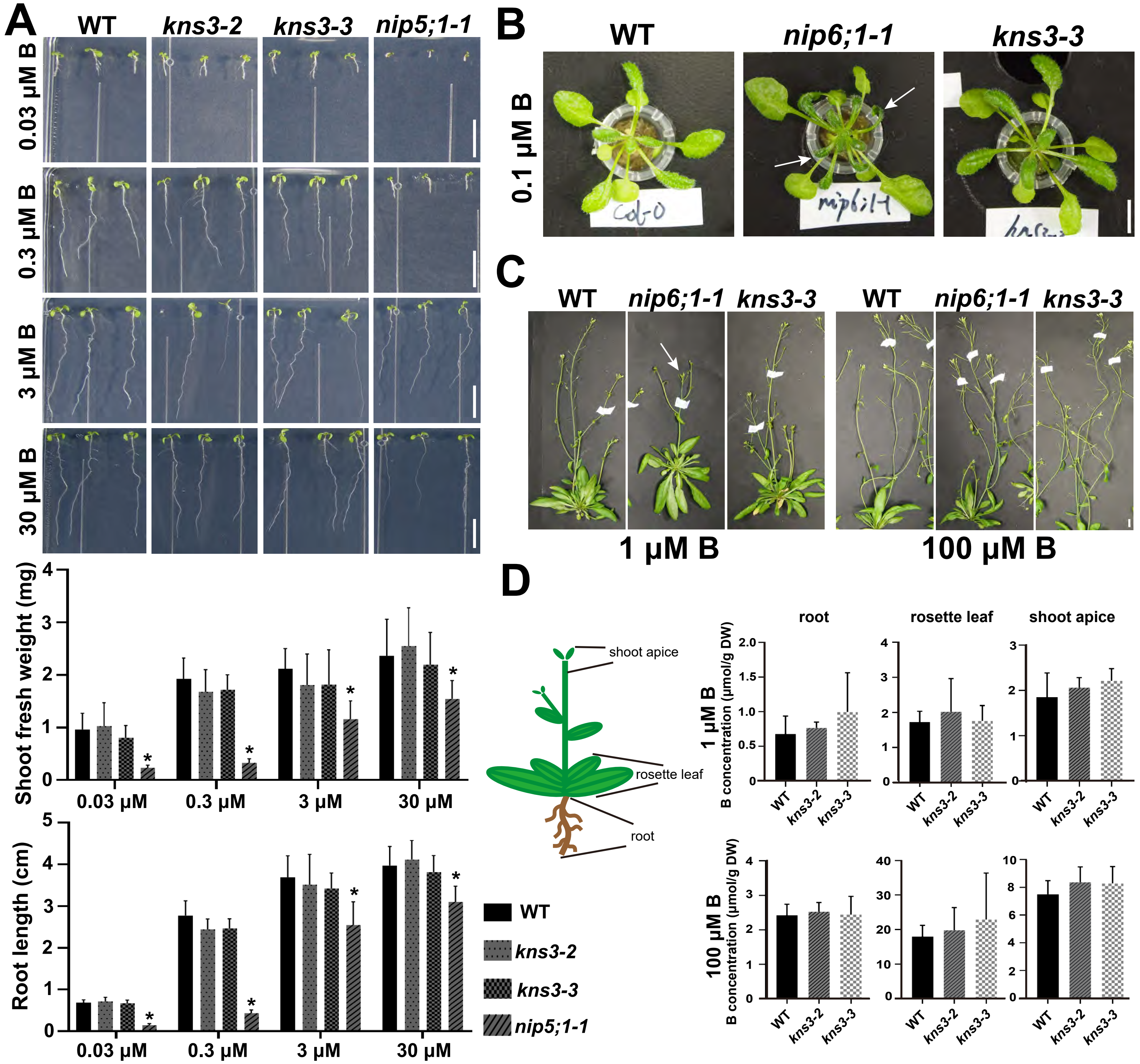

Fig. S3. Growth of *kns3* mutants under different B conditions.

(A) WT, *kns3-2*, *kns3-3,* and *nip5;1-1* were grown on solid medium contained 0.03, 0.3, 3, and 30 μM B for seven days. Fresh weights of shoots and root lengths were measured from 8–13 plants. Data represent the mean ± SD. The asterisks indicate mutants that showed significant differences to the WT (Dunnett' s test, *$P<0.01$). (B) WT, *kns3-3,* and *nip6;1-1* plants were grown hydroponically supplied with 0.1 μM B under a short-day condition (8 h/16 h light/dark cycle) for 30 days. Arrows indicate young rosette leaves with reduced expansion. (C) WT, *kns3-3,* and *nip6;1-1* plants were grown hydroponically supplied with 1 and 100 μM B under a long-day condition (16 h/8 h light/dark cycle) for 30 days. An arrow indicates the flowers of the main stem. (D) Boron concentrations in tissues of WT, *kns3-2,* and *kns3-3* plants grown hydroponically with 30 μM B for 10–12 days and then with 1 or 100 μM B for 15 days. Roots, rosette leaves, and shoot apices (1.5 cm from top) were harvested from three to four plants. Data represent the mean ± SD. No significant differences were observed (Mann-Whitney U-test, $P>0.05$). Scale bar=1 cm.

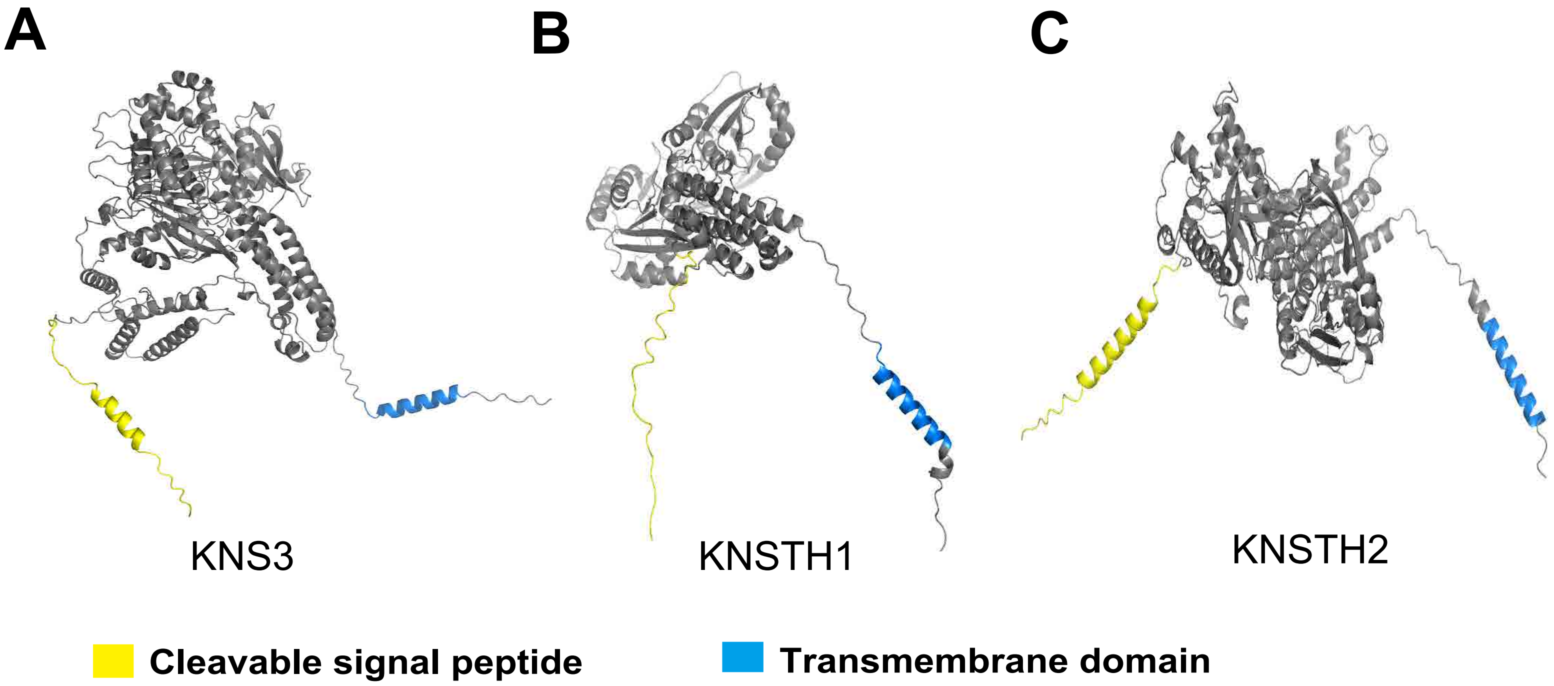

Fig. S4. Topologies of KNS3, KNSTH1, and KNSTH2.

Topologies of KNS3 (A), KNSTH1 (B), and KNSTH2 (C) as predicted by AlphaFold protein structure database.

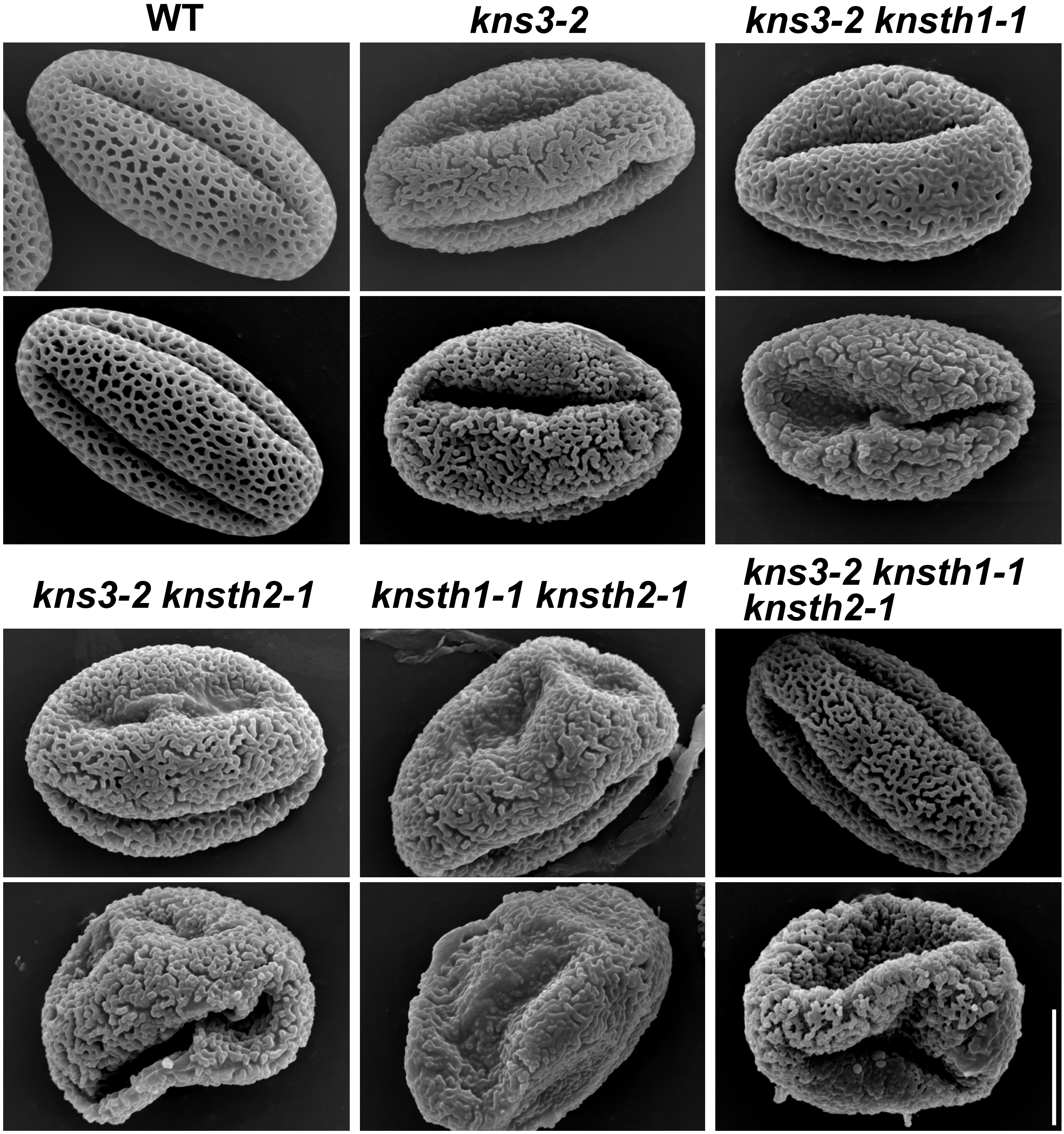

Fig. S5. Pollen structure in *kns3*, *knsth1*, and *knsth2* multiple mutants.

SEM images of pollens from WT, *kns3-2*, *kns3-2 knsth1-1, kns3-2 knsth2-1, knsth1-1 knsth2-1,* and *kns3-2 knsth1-1 knsth2-1* mutants. Plants were grown in pots with vermiculite supplied with 1/1000 diluted Hyponex solution (Hyponex Japan). Scale bar=10 μm.

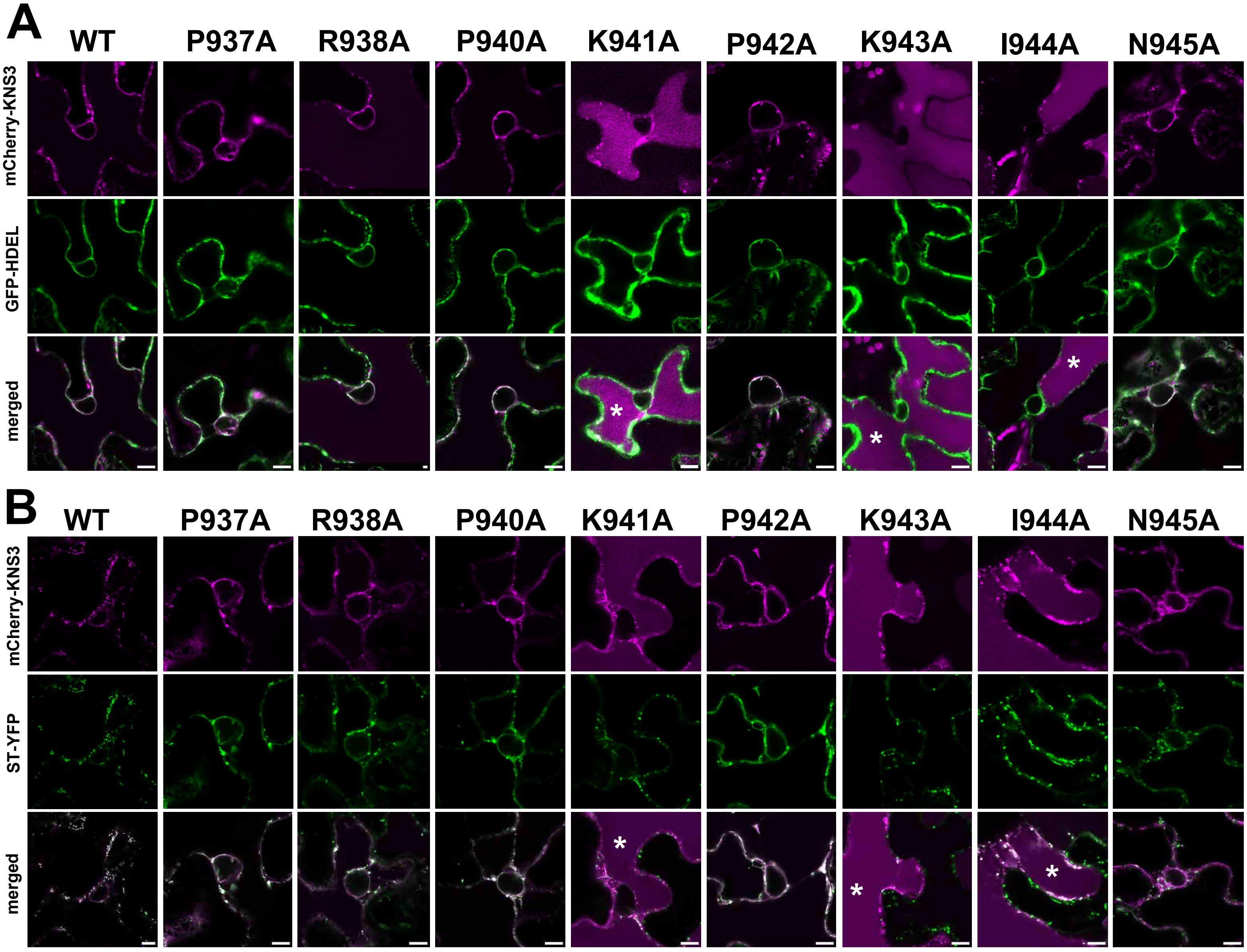

Fig. S6. K941, K943, and I944 in the C-terminal tail of KNS3 are important for its trafficking from the Golgi to the ER.

(A, B) mCherry-KNS3 WT and variants were introduced with GFP-HDEL (A) or ST-YFP (B) in *N.benthamiana* leaf epidermal cells by agroinfiltration. Asterisks indicate the vacuole. Scale bar=10 μm.

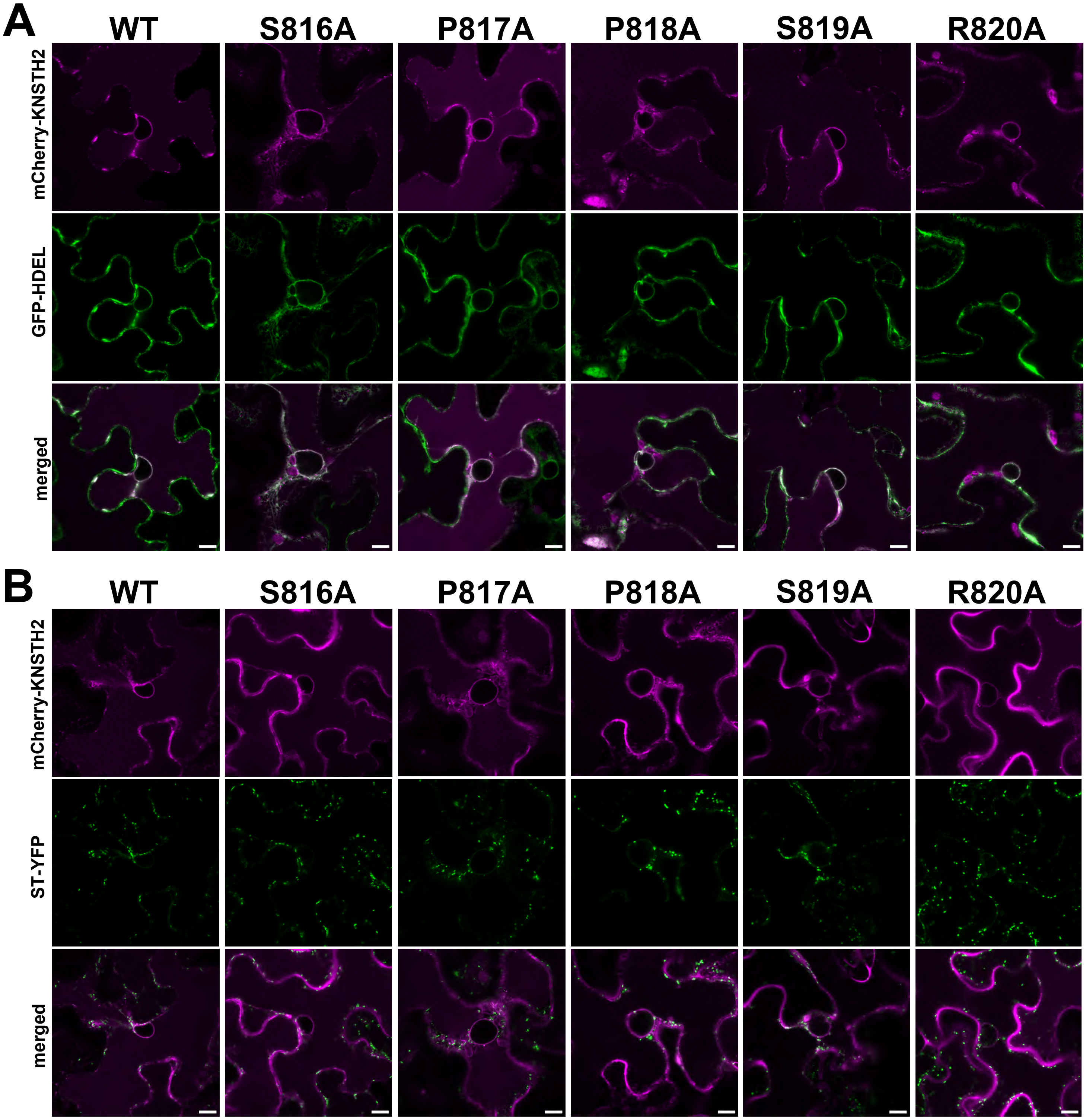

Fig. S7. Mutations in the C-terminal tail of KNSTH2 do not affect its ER localization.

(A, B) mCherry-KNSTH2 WT and variants were introduced with GFP-HDEL (A) or ST-YFP (B) in *N.benthamiana* leaf epidermal cells by agroinfiltration. Scale bar=10 μm.

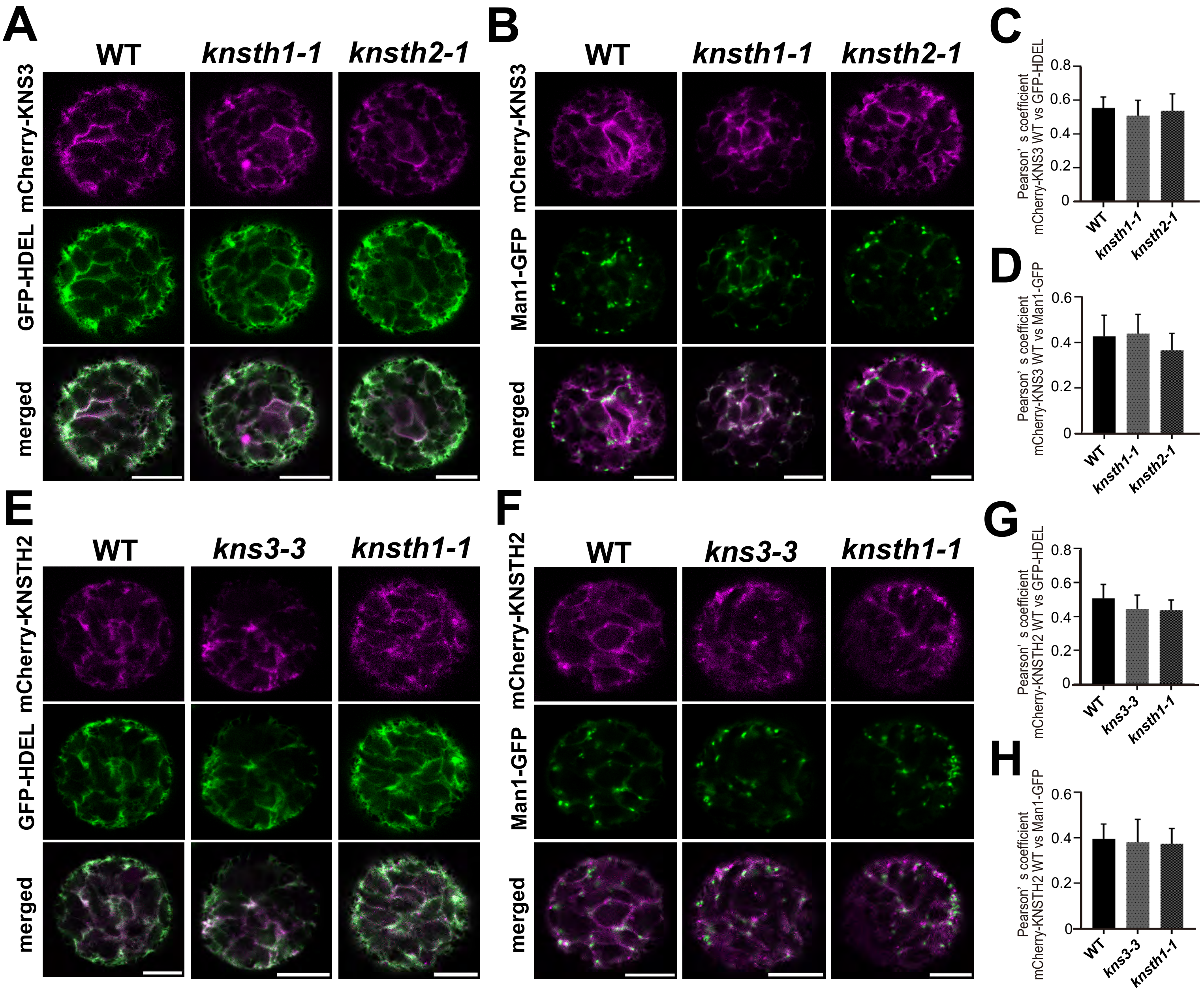

Fig. S8. Localization of mCherry-KNS3 and KNSTH2 is unchanged in single mutants of their homologs.

(A-D) mCherry-KNS3 and GFP-HDEL (A) or Man1-GFP (B) in protoplasts from WT (Col-0), *knsth1-1,* and *knsth2-1* Arabidopsis leaf mesophyll cells. Pearson' s coefficients of mCherry-KNS3 with GFP-HDEL (C) or Man1-GFP (D). Data represent the mean ± SD of 10 to 16 protoplasts. No significant differences were observed (Dunnett' s test, $P>0.05$). (E-H) mCherry-KNSTH2 and GFP-HDEL (E) or Man1-GFP (F) in protoplasts from WT (Col-0), *kns3-3,* and *knsth1-1* Arabidopsis leaf mesophyll cells. Pearson' s coefficients of mCherry-KNSTH2 with GFP-HDEL (G) or Man1-GFP (H). Data represent the mean ± SD of 10 to 11 protoplasts. No significant differences were observed (Dunnett' s test, $P>0.05$). Scale bar=10 μm.

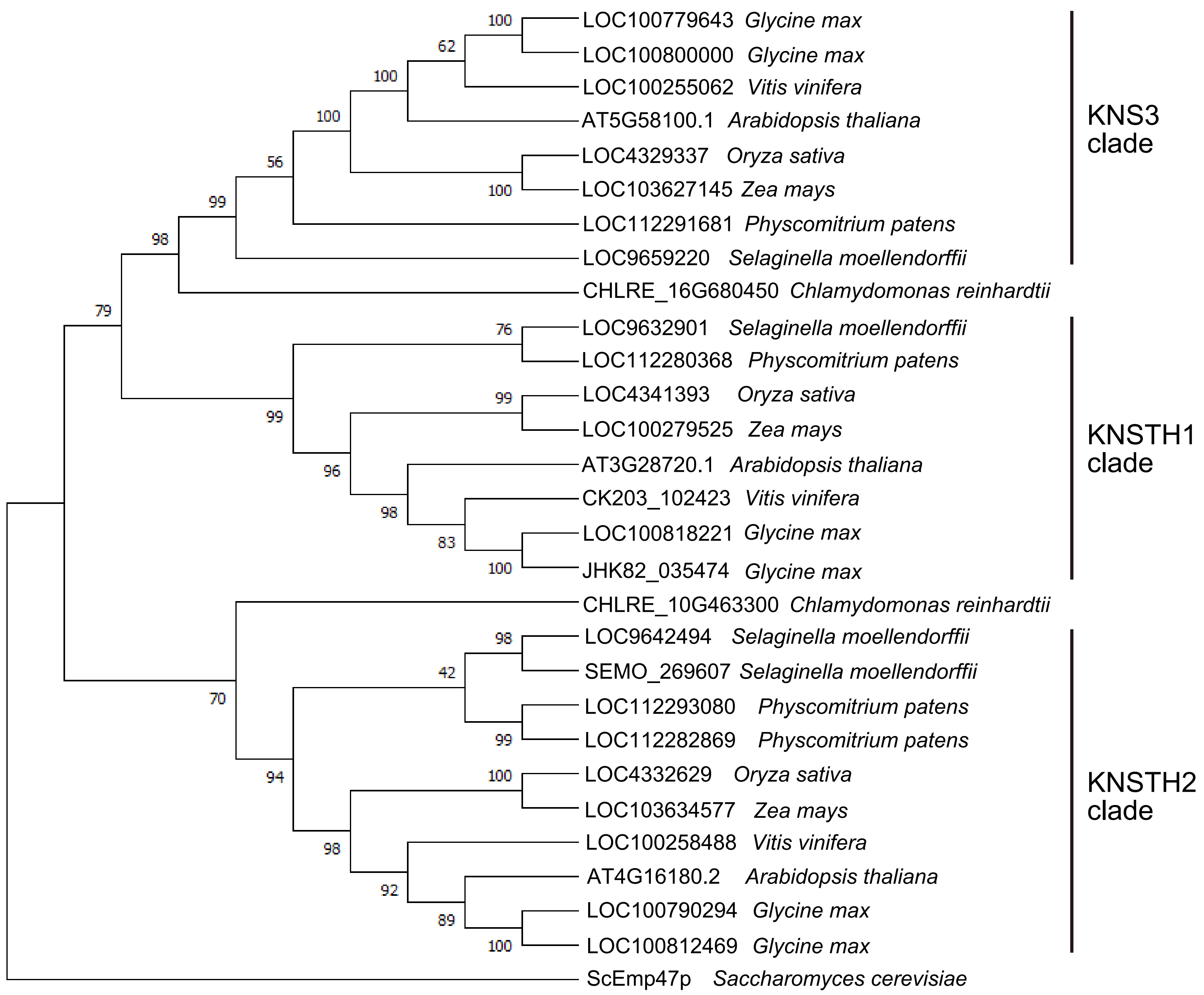

Fig. S9. Phylogenetic tree of KNS3 and its homologs.

Protein sequences of KNS3 homologs (from the National Center for Biotechnological Information Database) in different plant species were used for alignment, and a maximum likelihood-based tree was generated using the MEGA X program. Bootstrap testing was conducted with 1000 replicates. Values are indicated at branch nodes.

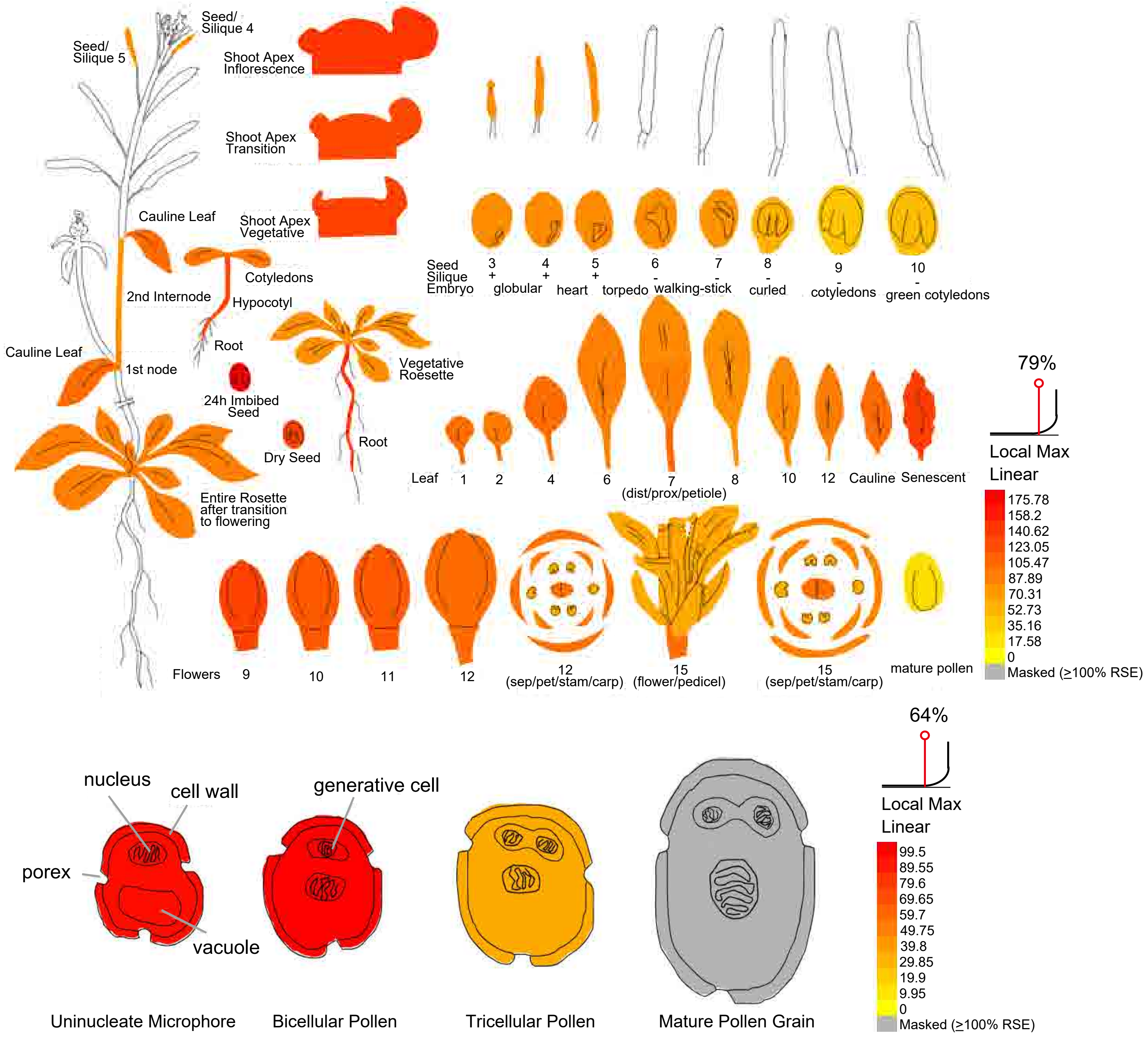

Fig. S10. Expression pattern of *KNS3*.

Expression patterns of *KNS3* in whole plants and pollen. Data were obtained from the ePlant database (https://bar.utoronto.ca/eplant/).

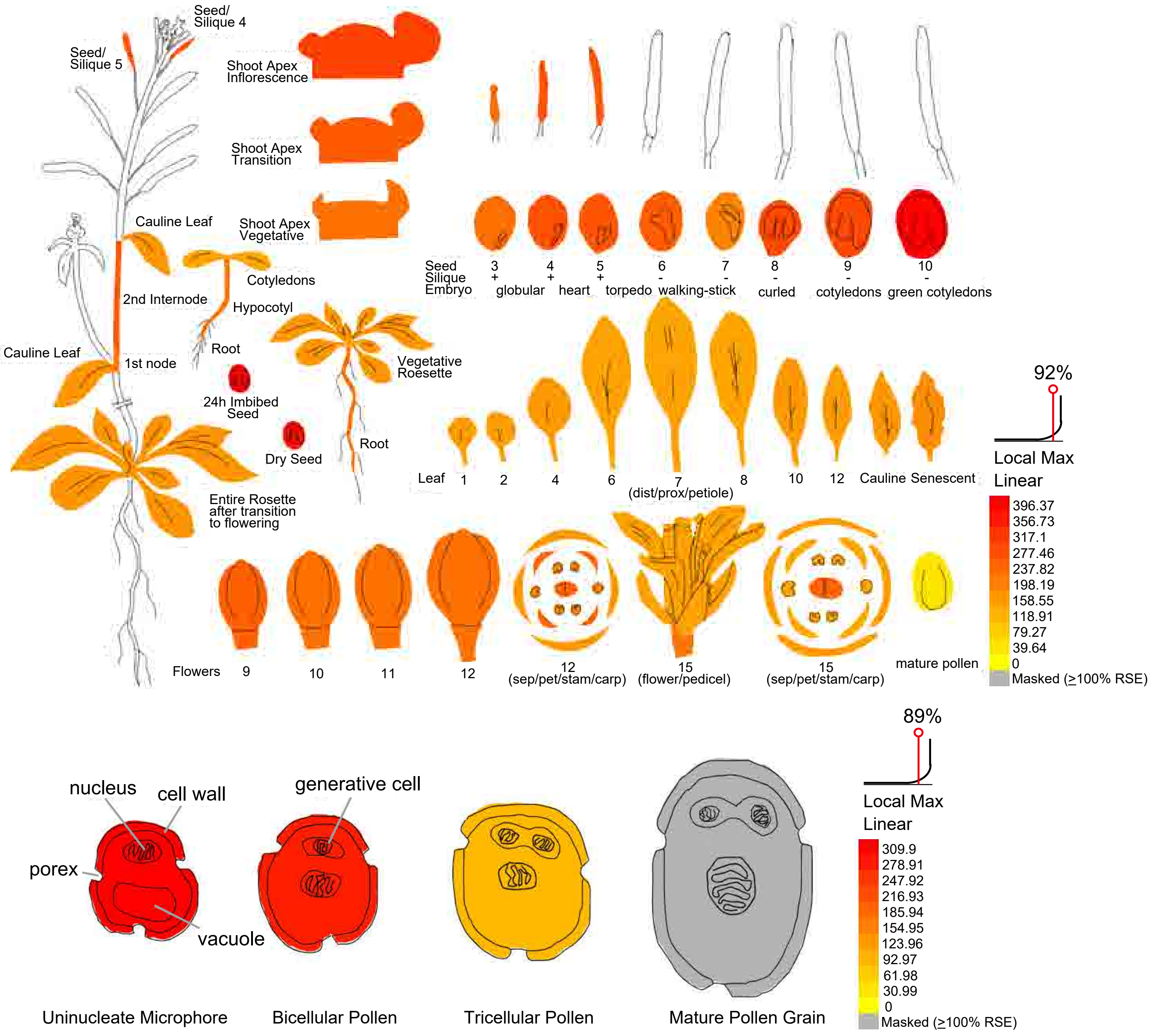

Fig. S11. Expression pattern of *KNSTH1*.

Expression patterns of *KNSTH1* in whole plants and pollen. Data were obtained from the ePlant database (https://bar.utoronto.ca/eplant/).

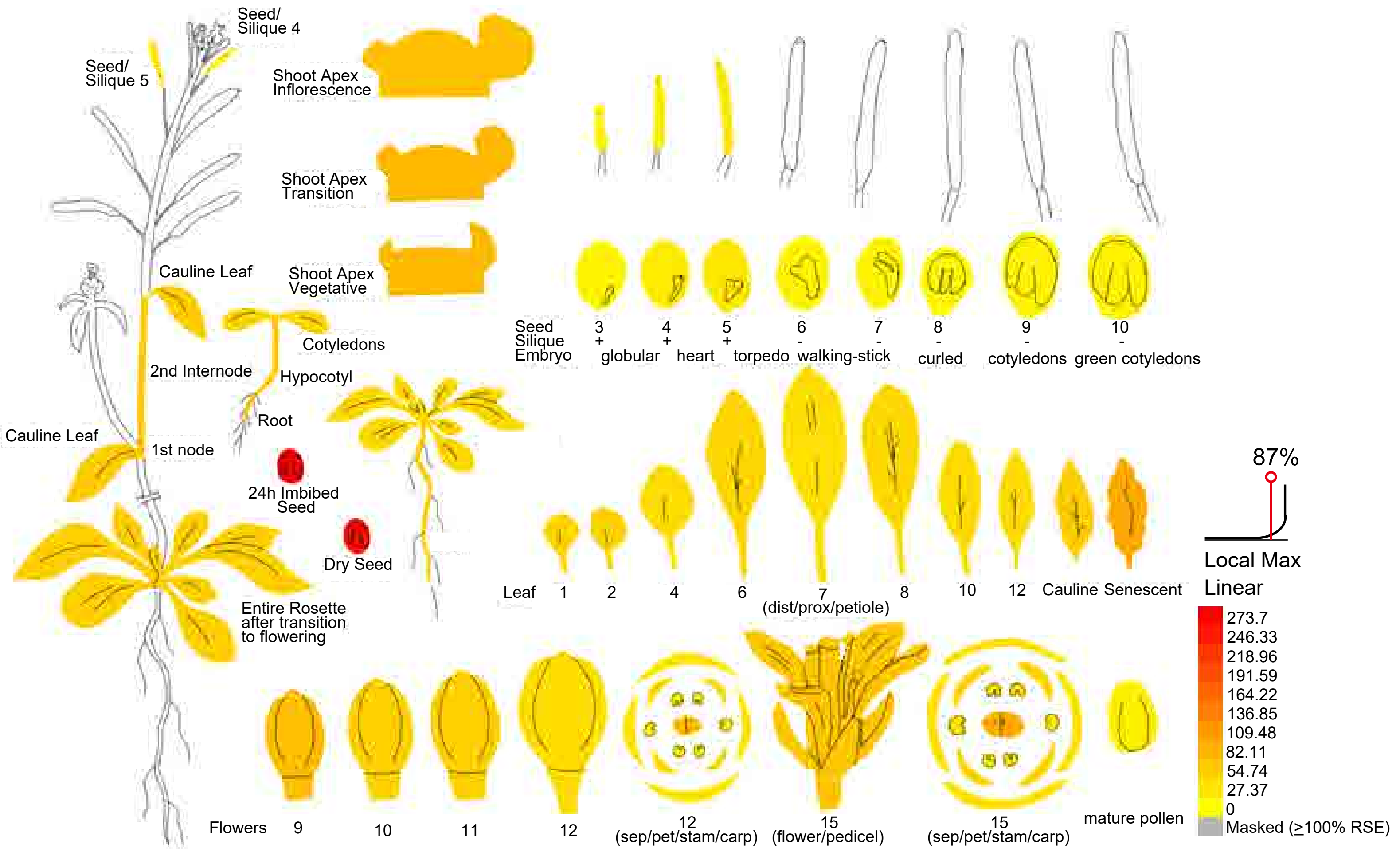

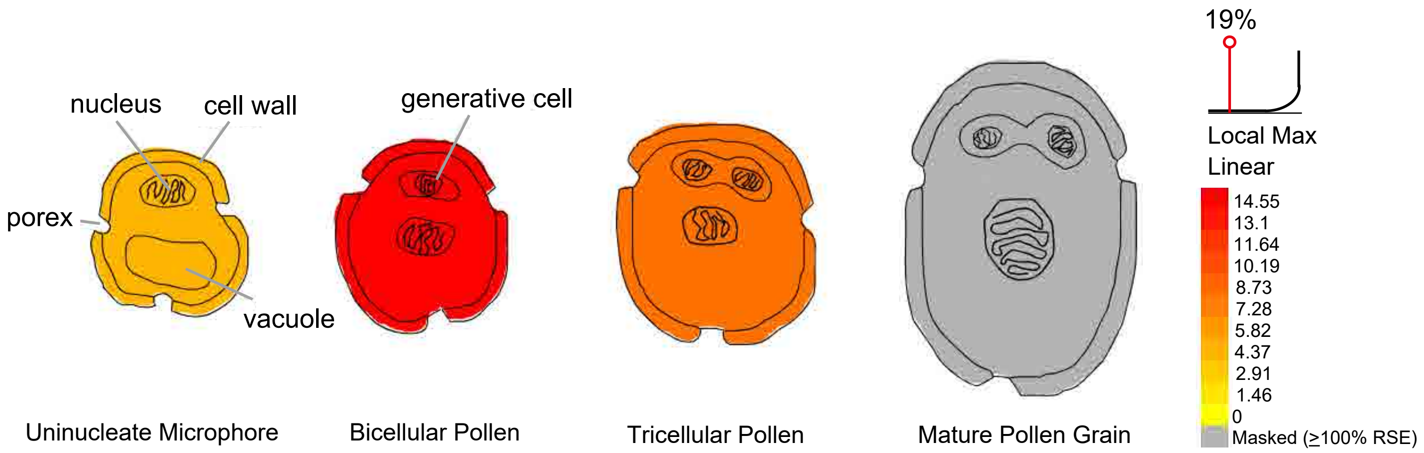

Fig. S12. Expression pattern of *KNSTH2*.

Expression patterns of *KNSTH2* in whole plants and pollen. Data were obtained from the ePlant database (https://bar.utoronto.ca/eplant/).

Clade	Accession	Species	Sequence	Position
KNS3 clade	AT5G58100.1	*Arabidopsis thaliana*	- - - - R P R A P K P K I N	945
	LOC100779643	*Glycine max*	- - - - R P R R P K P K I N	948
	LOC100800000	*Glycine max*	- - - - R P R R P K P K I N	956
	LOC100255062	*Vitis vinifera*	- - - - R P R R P K P K I N	938
	LOC4329337	*Oryza sativa*	- - - - R P R R P K P K I N	951
	LOC103627145	*Zea mays*	- - - - R P R R P K P K I N	953
	LOC9659220	*Selaginella moellendorffii*	- - - - - P R R L K P K I N	949
	LOC112291681	*Physcomitrium patens*	- - - - R R R R P K P K I N	953
KNSTH1 clade	AT3G28720.1	*Arabidopsis thaliana*	- K R D R L F R N K R K Q F	687
	LOC100818221	*Glycine max*	- R R D K L F R N K R K Q F	685
	JHK82_035474	*Glycine max*	- R R D K L F R N K R K Q F	686
	CK203_102423	*Vitis vinifera*	- K R D K L F R N K R K Q F	673
	LOC4341393	*Oryza sativa*	- K R D K L F R S K R K Q F	672
	LOC100279525	*Zea mays*	- K R D K L F R S K R K Q F	671
	LOC9632901	*Selaginella moellendorffii*	- R R E Q L F A S K R K R F	678
	LOC112280368	*Physcomitrium patens*	N K R D K F L V N K K K R F	676
KNSTH2 clade	AT4G16180.2	*Arabidopsis thaliana*	- - - - - - - - - S P P S R	820
	LOC100790294	*Glycine max*	- - - - - - - - - - S P V R	803
	LOC100812469	*Glycine max*	- - - - - - - - - - S P V R	803
	LOC100258488	*Vitis vinifera*	- - - - - - - - - - S P V R	809
	LOC4332629	*Oryza sativa*	- - - - - - - - - - S P V R	807
	LOC103634577	*Zea mays*	- - - - - - - - - - S P V R	804
	LOC9642494	*Selaginella moellendorffii*	- - - - - - - - - S G D K W	806
	SEMO_269607	*Selaginella moellendorffii*	- - - - - - - - - S G D K W	803
	LOC112293080	*Physcomitrium patens*	- - - - - - - - - - S S E R	820
	LOC112282869	*Physcomitrium patens*	- - - - - - - - - - S S E R	817

Fig. S13. Multiple alignments of the amino acid sequences of the C-terminal tail of KNS3 and its homologs.

Multiple alignments of the C-terminal tails of KNS3 homologs were performed using Clustal Omega. The conserved amino acids are highlighted in gray.

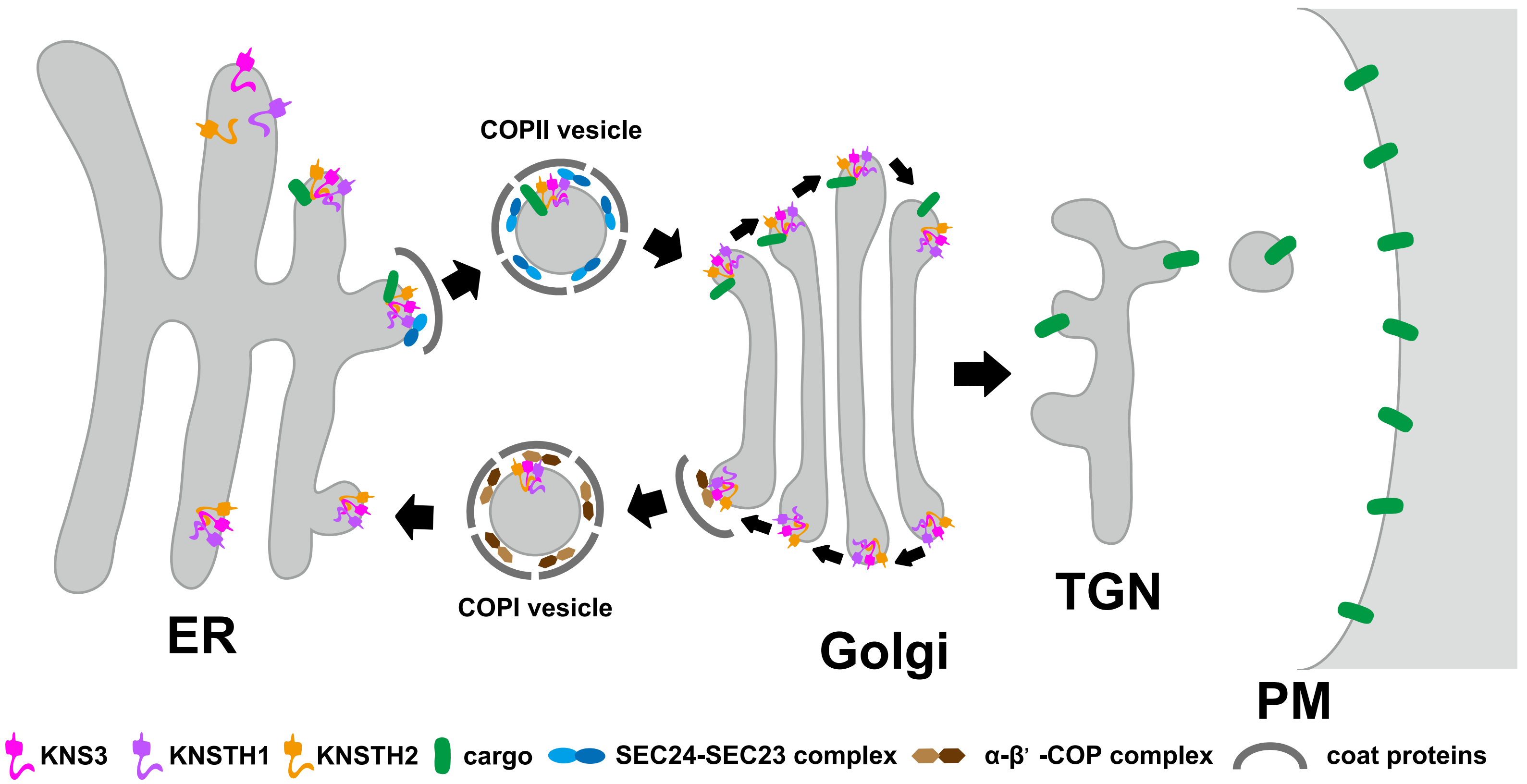

Fig. S14. A working hypothesis of the functions of KNS3, KNSTH1, and KNSTH2 in an ER-Golgi cargo-receptor complex.

In our hypothetical model, KNS3, KNSTH1, and KNSTH2 form a cargo-receptor complex that interacts with boric acid channels and other cargoes in the ER, packaging them into COPII vesicles for transport to the Golgi. In the Golgi, the cargo-receptor complex separates from the cargo and returns to the ER via COPI vesicles. Boric acid channels and other PM cargoes are transported from the Golgi apparatus to the trans-Golgi network, and then to the PM via the secretion pathway.

	Name	Location (Mbp)	Sequence		Product size Col-0/Ler (bp)
			forward	reverse	
Chromosome 1	map 1-3M	3	CGTGAACCCACTCGTTACATT	TGCATTTCAACTTTACCAACCA	248/204
	map 1-13.8M	13.8	ATGTTGGATTCAAGCACTTCC	AAGGTTCCGTCAGACGTG	200/156
	map 1-24.4M	24.4	TACTCATGCGGATGCGGTTA	TCTCCCTCCCTTTTCTTGCT	187/132
Chromosome 2	map 2-3.5M	3.5	ATCAACATCCGCAAAGTTCC	ACCTCCTTAGTCGCGTGAAA	383/327
	nga 168	16.3	GAGGACATGTATAGGAGCCTCG	TCGTCTACTGCACTGCCG	150/130
Chromosome 3	nga 162	4.6	CTCTGTCACTCTTTTCCTCTGG	CATGCAATTTGCATCTGAGG	110/85
	map 3-18M	18.2	TCGAGGACTTTTATTGATAGATTGAA	TGGATGAAAAGAAGGCAAGG	265/182
Chromosome 4	NGA8	5.6	TGGCTTTCGTTTATAAACATCC	GAGGGCAAATCTTTATTTCGG	154/198
Chromosome 5	2.5M	2.5	CCAAGACCAAAACCAAAACC	CATGCAATAGGCTTCGGAGT	247/173
	map 5-19.9M	19.9	TGACAACTTTGGGCAATTAAGA	CGCATGATGCATAGCAAAGT	246/209

Table S1. SSLP markers for rough mapping.

Primer name	Sequence (5’>3’)	Purpose
KNS3 (1-34aa) F	CTGATTAACACTCGAATGCGGAGATTCGGGGCT	KNS3 (1-34aa) for In-Fusion cloning
KNS3 (1-34aa) R	TCCTCCTCGCCCTTGCTCAATTGTGAAGCTCCATACGATAGA	
mCherry F	TTGAGCAAGGGCGAGGAG	mCherry for In-Fusion cloning
mCherry R	CTTGTACAGCTCGTCCATGCC	
KNS3 (35-945aa) F	GCATGGACGAGCTGTACAAGGGGAACCGGAAGACGGCGAA	KNS3 (35-945aa) for In-Fusion cloning
KNS3 (35-945aa) R	AGTTGGATATCTCGATCAGTTGATCTTTGGCTTAG	
KNSTH2 (1-36aa) F	CTGATTAACACTCGAGTCATGGAGTTGAGATCGG	KNSTH2 (1-36aa) for In-Fusion cloning
KNSTH2 (1-36aa) R	TCCTCCTCGCCCTTGCTCAATTGCTGAGCCGAGTCGGTA	
KNSTH2 (37-820aa) F	GCATGGACGAGCTGTACAAGCCTTTCCGTCGCGAGCCA	KNSTH2 (37-820aa) for In-Fusion cloning
KNSTH2 (37-820aa) R	AGTTGGATATCTCGACTAGCGAGAAGGAGGAGA	
P937A F	TTAAGGGCTAGAGCTCCTAAGCCAAAG	Introduction of mCherry-KNS3 P937A mutation
P937A R	AGCTCTAGCCCTTAAGACAGCGTAAAG	
R938A F	AGGCCAGCTGCTCCTAAGCCAAAGATC	Introduction of mCherry-KNS3 R938A mutation
R938A R	AGGAGCAGCTGGCCTTAAGACAGCGTA	
P940A F	AGAGCTGCTAAGCCAAAGATCAACTGA	Introductio

P940A R	TGGCTTAGCAGCTCTTGGCCTTAAGAC	n of mCherry-KNS3 P940A mutation
K941A F	GCTCCTGCTCCAAAGATCAACTGATCG	Introduction of mCherry-KNS3 K941A mutation
K941A R	CTTTGGAGCAGGAGCTCTTGGCCTTAA	
P942A F	CCTAAGGCTAAGATCAACTGATCGAGA	Introduction of mCherry-KNS3 P942A mutation
P942A R	GATCTTAGCCTTAGGAGCTCTTGGCCT	
K943A F	AAGCCAGCTATCAACTGATCGAGATAT	Introduction of mCherry-KNS3 K943A mutation
K943A R	GTTGATAGCTGGCTTAGGAGCTCTTGG	
I944A F	CCAAAGGCTAACTGATCGAGATATCCA	Introduction of mCherry-KNS3 I944A mutation
I944A R	TCAGTTAGCCTTTGGCTTAGGAGCTCT	
N945A F	AAGATCGCTTGATCGAGATATCCAACT	Introduction of mCherry-KNS3 N945A mutation
N945A R	CGATCAAGCGATCTTTGGCTTAGGAGC	
S816A F	TTCTCTGCTCCTCCTTCTCGCTAGTCG	Introductio

S816A R	AGGAGGAGCAGAGAAGAAAATGACAAG	n of mCherry-KNSTH2 S816A mutation
P817A F	TCTTCTGCTCCTTCTCGCTAGTCGAGA	Introduction of mCherry-KNSTH2 P817A mutation
P817A R	AGAAGGAGCAGAAGAGAAGAAAATGAC	
P818A F	TCTCCTGCTTCTCGCTAGTCGAGATAT	Introduction of mCherry-KNSTH2 P818A mutation
P818A R	GCGAGAAGCAGGAGAAGAGAAGAAAAT	
S819A F	CCTCCTGCTCGCTAGTCGAGATATCCA	Introduction of mCherry-KNSTH2 S819A mutation
S819A R	CTAGCGAGCAGGAGGAGAAGAGAAGAA	
R820A F	CCTTCTGCTTAGTCGAGATATCCAACT	Introduction of mCherry-KNSTH2 R820A mutation
R820A R	CGACTAAGCAGAAGGAGGAGAAGAGAA	
LBb1.3	ATTTTGCCGATTTCGGAAC	Genotyping for SALK T-DNA mutant
SALK_041228 LP	TCAGTGCATACATATGCTGGC	Genotyping for *kns3-2*
SALK_041228	ACCATCAGGCTGTTGACATTC	

RP		
SALK_061320 LP	AAAGGAGCCAACCTTGAGAAG	Genotyping for *kns3-3*
SALK_061320 RP	AAAGAAGCCTTTCCTTGATGC	
SALK_027378 LP	GTTCAAGAAAACGTCAGACGC	Genotyping for *knsth1-1*
SALK_027378 RP	CGTCAAGGTGGAGAGAGTGAG	
SALK_106609 LP	CATCGGAGACTCTTTCCCTTC	Genotyping for *knsth1-2*
SALK_106609 RP	GCCCGTTGCAAGTATAATCAC	
LB3	TAGCATCTGAATTTCATAACCAATCTCGATACAC	Genotyping for SAIL T-DNA mutant
SAIL_731_H03 LP	AGGTGTAGGCTAGCGAGAAGG	Genotyping for *knsth2-1*
SAIL_731_H03 RP	ATGCTGTCCCATCACAGGTAC	
SAIL_670_H01 LP	TAGCCGATGTAGATCCAATGC	Genotyping for *knsth2-3*
SAIL_670_H01 RP	ACCAGCCACAAGTATTCCTCC	

Table S2. Primers used in this research.

		localization			
		in Arabidopsis protoplasts		in tobacco leaf cells	
KNS3	WT	ER, Golgi	(Figs 6, 8)	ER, Golgi	(Fig. 6, Fig. S6)
	P937A	ER, Golgi	(Fig. 8)	ER, Golgi	(Fig. S6)
	R938A	ER, Golgi		ER, Golgi	
	P940A	ER, Golgi		ER, Golgi	
	K941A	ER, Golgi, vacuole		Golgi, vacuole	
	P942A	ER, Golgi		ER, Golgi	
	K943A	ER, Golgi, vacuole		Golgi, vacuole	
	I944A	Golgi		Golgi, vacuole	
	N945A	ER, Golgi		ER, Golgi	
KNSTH2	WT	ER	(Figs 7, 9)	ER	(Fig. 7, Fig. S7)
	S816A	ER	(Fig. 9)	ER	(Fig. S7)
	P817A	ER		ER	
	P818A	ER		ER	
	S819A	ER		ER	
	R820A	ER		ER	

	in Arabidopsis protoplasts		
	WT	*knsth1-1*	*knsth2-1*
KNS3 WT	ER, Golgi (Figs 6, 8, Fig. S8)	ER, Golgi (Fig. S8)	ER, Golgi (Fig. S8)
KNSTH2 WT	WT	*kns3-3*	*knsth1-1*
	ER (Figs 7, 9, Fig. S8)	ER (Fig. S8)	ER (Fig. S8)
KNS3 I944A	WT	*knsth1-1/knsth1-2*	*knsth2-1/knsth2-3*
	Golgi (Figs 8, 10)	ER, Golgi (Fig. 10)	ER, Golgi (Fig. 10)

Table S3. Summary of the localization of mCherry-KNS3 and mCherry-KNSTH2 in Arabidopsis protoplasts and tobacco leaf cells.

Organism	Name	Protein length (aa)	NCBI protein number
Arabidopsis thaliana	AT5G58100.1	945	NP_001318823.1
	AT4G16180.2	820	NP_001154242.2
	AT3G28720.1	687	NP_189514.1
Glycine max	LOC100779643	948	XP_003516388.1
	LOC100800000	956	XP_003532318.1
	LOC100818221	685	XP_003554877.2
	JHK82_035474	686	KAG5112205.1
	LOC100790294	803	XP_003528615.1
	LOC100812469	803	XP_003550564.1
Vitis vinifera	LOC100255062	938	XP_010655027.1
	CK203_102423	673	RVW26491.1
	LOC100258488	809	XP_002273166.2
Oryza sativa	LOC4329337	951	XP_015624632.1
	LOC4341393	672	XP_015644546.1
	LOC4332629	807	XP_015632688.1
Zea mays	LOC103627145	953	XP_008645675.1
	LOC100279525	671	XP_001145995.2
	LOC103634577	804	XP_008655047.1
Selaginella moellendorffii	LOC9659220	949	XP_024530236.1
	LOC9632901	678	XP_002991685.1
	LOC9642494	806	XP_024516482.1
	SEMO_269607	803	EFJ08873.1
Physcomitrium patens	LOC112291681	953	XP_024395234.1
	LOC112280368	676	XP_024371568.1
	LOC112293080	820	XP_024397913.1
	LOC112282869	817	XP_024376759.1
Chlamydomonas reinhardtii	CHLRE_16G680450	1079	XP_042916023.1
	CHLRE_10G463300	1012	XP_042920595.1

Table S4. List of proteins collected using BLAST in the NCBI database.
